# Gut microbiota plasticity is correlated with sustained weight loss on a low-carb or low-fat dietary intervention

**DOI:** 10.1038/s41598-020-58000-y

**Published:** 2020-01-29

**Authors:** Jessica A. Grembi, Lan H. Nguyen, Thomas D. Haggerty, Christopher D. Gardner, Susan P. Holmes, Julie Parsonnet

**Affiliations:** 1 Department of Civil and Environmental Engineering, Stanford University, 318 Campus Drive E250 Clark Center, Stanford, CA 94305 United States; 2 Department of Medicine, Stanford University School of Medicine, 291 Campus Drive, Stanford, CA 94305 United States; 3 Institute for Computational and Mathematical Engineering, Stanford University, 475 Via Ortega, Stanford, CA 94305 United States; 4 Stanford Prevention Research Center, Department of Medicine, Stanford University School of Medicine, 1265 Welch Road, Stanford, CA 94305 United States; 5 Department of Statistics, Stanford University, 390 Serra Mall, Stanford, CA 94305 United States; 6 Department of Health Research and Policy, Stanford University School of Medicine, 150 Governor’s Ln, Stanford, CA 94305 United States

**Keywords:** Microbiome, Obesity

## Abstract

While low-carbohydrate and low-fat diets can both lead to weight-loss, a substantial variability in achieved long-term outcomes exists among obese but otherwise healthy adults. We examined the hypothesis that structural differences in the gut microbiota explain a portion of variability in weight-loss using two cohorts of obese adults enrolled in the Diet Intervention Examining The Factors Interacting with Treatment Success (DIETFITS) study. A total of 161 pre-diet fecal samples were sequenced from a discovery cohort (n = 66) and 106 from a validation cohort (n = 56). An additional 157 fecal samples were sequenced from the discovery cohort after 10 weeks of dietary intervention. We found no specific bacterial signatures associated with weight loss that were consistent across both cohorts. However, the gut microbiota plasticity (i.e. variability), was correlated with long-term (12-month) weight loss in a diet-dependent manner; on the low-fat diet subjects with higher pre-diet daily plasticity had higher sustained weight loss, whereas on the low-carbohydrate diet those with higher plasticity over 10 weeks of dieting had higher 12-month weight loss. Our findings suggest the potential importance of gut microbiota plasticity for sustained weight-loss. We highlight the advantages of evaluating kinetic trends and assessing reproducibility in studies of the gut microbiota.

## Introduction

The global obesity pandemic has claimed one in three American adults and prevalences continue to rise in many other countries as well^1^. Obesity comorbidities (e.g. cardiovascular diseases, cancer, diabetes, and other chronic conditions) cost the US $8.65 billion/yr from loss of productivity due to absenteeism alone^2^. The personal, social and economic costs provide an urgent need for consistently effective, and possibly more personalized, weight-reduction therapies.

Different dietary interventions, such as low-carbohydrate (low-carb) and low-fat diets, can lead to weight loss, but not always; there remains substantial variability in diet success outcomes among obese, but otherwise healthy, adults^3^. Additionally, adherence to dietary intervention strategies has remained a major challenge, despite the clear dose-response relationship with weight loss for individuals on both low-carb and low-fat diets^4,5^. Thus, practitioners have begun to look for individual characteristics (e.g. physiological attributes, cultural or lifestyle characteristics, food preferences, etc.) that could influence an individual’s sustained weight loss, possibly by improving adherence to a specific dietary regime.

Gut microbiota are highly individualized and intricately involved with the quantity and quality of nutrients extracted from our diets, with direct implications for obesity^6–9^. Microbial metabolites and proteins are known to communicate with the host to influence appetite control^10–12^. For example, proteins from gut *E. coli* modulate appetite by interacting with antigens involved in host satiety signaling^13^. Byproducts of microbial fermentation (e.g. butyrate and propionate) also stimulate gut hormones that reduce food intake^14,15^. Studies assessing inter-individual variability in gut microbiota alterations in response to dietary interventions are limited by short-term (<12 weeks) dietary modifications or measurement of metabolic outcomes (e.g. plasma glucose, triglycerides, insulin, cholesterol) rather than weight loss^16^. Two recent studies on overweight Danish adults investigated the relationship between baseline microbiota *Prevotella*/*Bacteroides* (P/B) ratio and found that individuals with high P/B ratio ( > 0.01) lost more weight and body fat after 6 months on a high-fiber dietary intervention than individuals with low P/B ratio^17,18^. No studies have investigated the impact of microbiota on adherence to a dietary intervention.

The DIETFITS study^19,20^ was a randomized trial of 609 adults designed to elucidate predisposing individual characteristics – genotype, insulin-glucose dynamics, physiological and psychosocial attributes – that contribute to successful 12-month weight loss on *ad libitum* diets designed to be lower in carbs or fat. Within a population subset of DIETFITS, we explored whether attributes of the gut microbiota predisposed individuals to successful 12-month weight loss. Identifying pre-diet features of the gut microbiota that can predict adherence and/or success on a specific diet might permit personalization of dietary intervention strategies to maximize weight loss.

## Results

### Subject demographics, weight loss and sequencing statistics

We recruited subjects from two cohorts of obese adults enrolled in the DIETFITS randomized trial of low-carb and low-fat diets. The cohorts were enrolled approximately six months apart, allowing one to be used for discovery and the second for validation. From each of these cohorts, individuals who a) provided fecal samples prior to initiating the intervention that passed quality filtering (>10,000 high-quality 16S rRNA sequences) and b) completed the one-year intervention were included in our study.

The discovery cohort included 66 subjects, of whom 32 (22 female) were randomized to the low-carb diet and 34 (17 female) to the low-fat diet. These 66 subjects provided fecal samples on three consecutive days prior to starting the diet plus three additional daily samples 10 weeks after diet initiation. A sequencing depth of 73, 659 ± 33, 380 reads per sample was obtained from 318 fecal samples (161 pre-diet; 157 at 10 weeks). Subject characteristics and dietary information can be found in Table 1. Over the course of the 12-month intervention, subjects on the low-carb diet restricted carbs to an average of 22.6 ± 10.3% of their daily kilocalories (kcals) and lost 8.4 ± 7.7% of their starting weight whereas those on the low-fat diet restricted fats to an average of 25.3 ± 5.7% of daily kcals and lost 6.3 ± 7.7% of their starting weight. Previous definitions of long-term weight loss success^21,22^ were used to categorize subjects based on the percentage of baseline weight lost at 12 months: 20 were unsuccessful (US: < 3% weight loss), 25 were moderately successful (MS: 3 − 10% weight loss), and 21 were very successful (VS: > 10% weight loss).

**Table 1:** Subject characteristics and dietary intake for discovery and validation cohorts.

	Low-carb		Low-fat	
	Discovery (n = 32)	Validation (n = 31)	Discovery (n = 34)	Validation (n = 25)
Sex, n (%)				
Female	22 (68.8)	25 (80.6)	17 (50)	19 (76)
Age, yr (SD)	43.1 (6)	42.5 (6)	40.5 (6.9)	39.6 (6)
Race/ethnicity, n (%)				
White	25 (78.1)	23 (74.2)	25 (73.5)	16 (64)
Hispanic	4 (12.5)	5 (16.1)	7 (20.6)	4 (16)
Asian	3 (9.4)	2 (6.5)	2 (5.9)	3 (12)
Other	0 (0)	1 (3.2)	0 (0)	2 (8)
Baseline weight, kg (SD)	92.9 (16.8)	90 (13.9)	96.1 (12.4)	92.6 (12.7)
Baseline body fat, % (SD)	38 (5.8)	38.4 (6.4)	34.7 (7)	37.4 (5.9)
Body mass index, kg/m^2^ (SD)	33.4 (3.7)	32.8 (3.5)	33.2 (3.3)	33.2 (3.5)
Metabolic syndrome, n (%)	11 (34.4)	7 (22.6)	7 (20.6)	7 (28)
Weight loss^*a*^, % (SD)	8.4 (7.7)	4.8 (6.2)	6.3 (7.7)	5.4 (7.6)
Weight loss^*a*^ success category, n (%)				
US	9 (28.1)	14 (45.2)	11 (32.4)	10 (40)
MS	10 (31.3)	11 (35.5)	15 (44.1)	7 (28)
VS	13 (40.6)	6 (19.3)	8 (23.5)	8 (32)
**Dietary Intake**				
Fat, % kcal (SD)				
Baseline	38.9 (6.1)	37.3 (6.4)	36.8 (6.5)	36.1 (5.2)
3 months	57 (9.1)	48.3 (8.7)	21.4 (7.2)	24.9 (7.5)
6 months	53.6 (9.1)	44.9 (7.5)	25.6 (7.6)	27.8 (9.1)
12 months	47.5 (10)	43.2 (8.4)	28.6 (8.2)	31.3 (7.9)
Carbohydrates, % kcal (SD)				
Baseline	44.6 (7.6)	45.9 (6.6)	46.4 (7)	46.8 (6.5)
3 months	17.6 (11)	27.7 (10.5)	59 (9.1)	54.4 (9.3)
6 months	22.1 (11.7)	31.4 (10.4)	55.6 (8)	52.3 (9.6)
12 months	27.3 (12.5)	35.4 (10.2)	53.3 (8.1)	48.1 (8.5)
Protein, % kcal (SD)				
Baseline	16.6 (3.4)	16.8 (2.9)	16.8 (3.4)	17.1 (3.4)
3 months	25.4 (6)	24 (6.1)	19.5 (4.6)	20.7 (5.4)
6 months	24.3 (5.2)	23.6 (6.8)	18.7 (4.2)	19.9 (5)
12 months	25.2 (7.1)	21.4 (4.9)	18.1 (3.9)	20.5 (4.5)
Total fiber, g/1000 kcal (SD)				
Baseline	9.9 (3.4)	10.8 (2.9)	10.5 (4.4)	12.2 (4)
3 months	9 (3.1)	15.3 (27.5)	20.1 (7.3)	15.6 (3.9)
6 months	9.2 (4.3)	11.6 (4.1)	16.1 (5.9)	15.5 (5.1)
12 months	11.4 (3.9)	11.5 (4.8)	14.6 (5.7)	13.4 (4)
Soluble fiber, g/1000 kcal (SD)				
Baseline	2.8 (0.8)	3.3 (1)	3.4 (1.5)	3.7 (1.3)
3 months	2.5 (1.4)	3.1 (1.4)	5.3 (1.8)	4.6 (1.5)
6 months	2.5 (1.4)	3.7 (2)	4.7 (1.9)	4.3 (1.4)
12 months	2.9 (1.1)	3.4 (1.5)	4.3 (1.5)	3.9 (1.1)
Insoluble fiber, g/1000 kcal (SD)				
Baseline	7 (3)	7.4 (2.3)	7.1 (3)	8.3 (2.8)
3 months	6.5 (2.4)	7.5 (3.2)	14.8 (5.9)	10.9 (3.1)
6 months	6.6 (3.4)	7.8 (2.6)	11.5 (4.7)	11.1 (4)
12 months	8.5 (3.4)	8 (3.8)	10.2 (4.5)	9.4 (3.3)
Dietary adherence^*b*^, % kcals (SD)	77.4 (10.3)	68.5 (9.1)	74.7 (5.7)	72.3 (7.4)
Dietary change^*c*^, % kcals (SD)	21.9 (10.5)	14.4 (10.4)	11.4 (7.3)	8.4 (6.1)

^*a*^Measured after 12 months of dietary intervention.

^*b*^Portion of diet from non-restricted foods. Carbohydrate restriction for subjects on low-carb diet; fat restriction for subjects on low-fat diet. Averaged over 3-, 6-, and 12-month dietary recalls.

^*c*^Reduction in restricted foods from baseline levels. Averaged over 3-, 6-, and 12-month dietary recalls.

The validation cohort was comprised of 56 subjects: 31 (25 female) on the low-carb and 25 (19 female) on the low-fat diet. Subject characteristics were comparable between the two cohorts (Table 1), except for the low-carb diet where the percent weight lost at 12 months was significantly lower in the validation cohort (4.8 ± 6.2% compared to 8.4 ± 7.7% in the discovery cohort, Welch’s t-test *p* = 0.045) as well as dietary adherence (68.5 ± 9.1% compared to 77.4 ± 10.3% in discovery cohort, Welch’s t-test *p* = 0.0005). Subjects were classified to weight loss success groups, as described above, with the following distribution: 24 US, 18 MS, and 14 VS. From these “validation” subjects, two fecal samples were collected prior to the start of the dietary intervention at a median of 12 (IQR 7, 25) days apart. Samples meeting quality criteria (n = 106) had a mean sequencing depth of 70, 041 ± 15, 664 reads per sample.

Each diet resulted in lower reported consumption of the restricted component (fats or carbs) compared to baseline (Table 1) and overall, subjects with VS long-term weight loss reduced fats or carbs more than US subjects (Supplementary Table S1). Given the observed sex-specific differences in correlations between reported adherence and weight loss in the larger DIETFITS study population, we conducted sub-group analyses on dietary adherence for men and women using two measures: dietary adherence (i.e. the % of total kcals consumed from non-restricted foods) and dietary change (i.e. the difference in percentage of total daily kcals consumed from restricted foods between baseline and during the dietary intervention). Across both cohorts, individuals with higher reported dietary adherence or dietary change did not always have higher weight loss (Supplementary Fig. S1). No significant differences in dietary adherence were noted between different success groups for men on the low-carb diet (Kruskal-Wallis *p* = 0.082) or women on the low-fat diet (*p* = 0.34). However, VS male subjects on the low-fat diet reported significantly higher dietary adherence than MS or US males on the same diet (pairwise Wilcoxon rank sum test between groups adjusted for multiple corrections with Benjamimi Hochberg procedure, *p* = 0.01 for both comparisons noted), and VS women on the low-carb diet reported higher diet adherence than US females on the same diet (*p* = 0.016). Similar results were found for dietary change (Supplementary Fig. S1b).

### Pre-diet gut microbial community composition does not predict 12-month weight loss success

**Figure 1:**
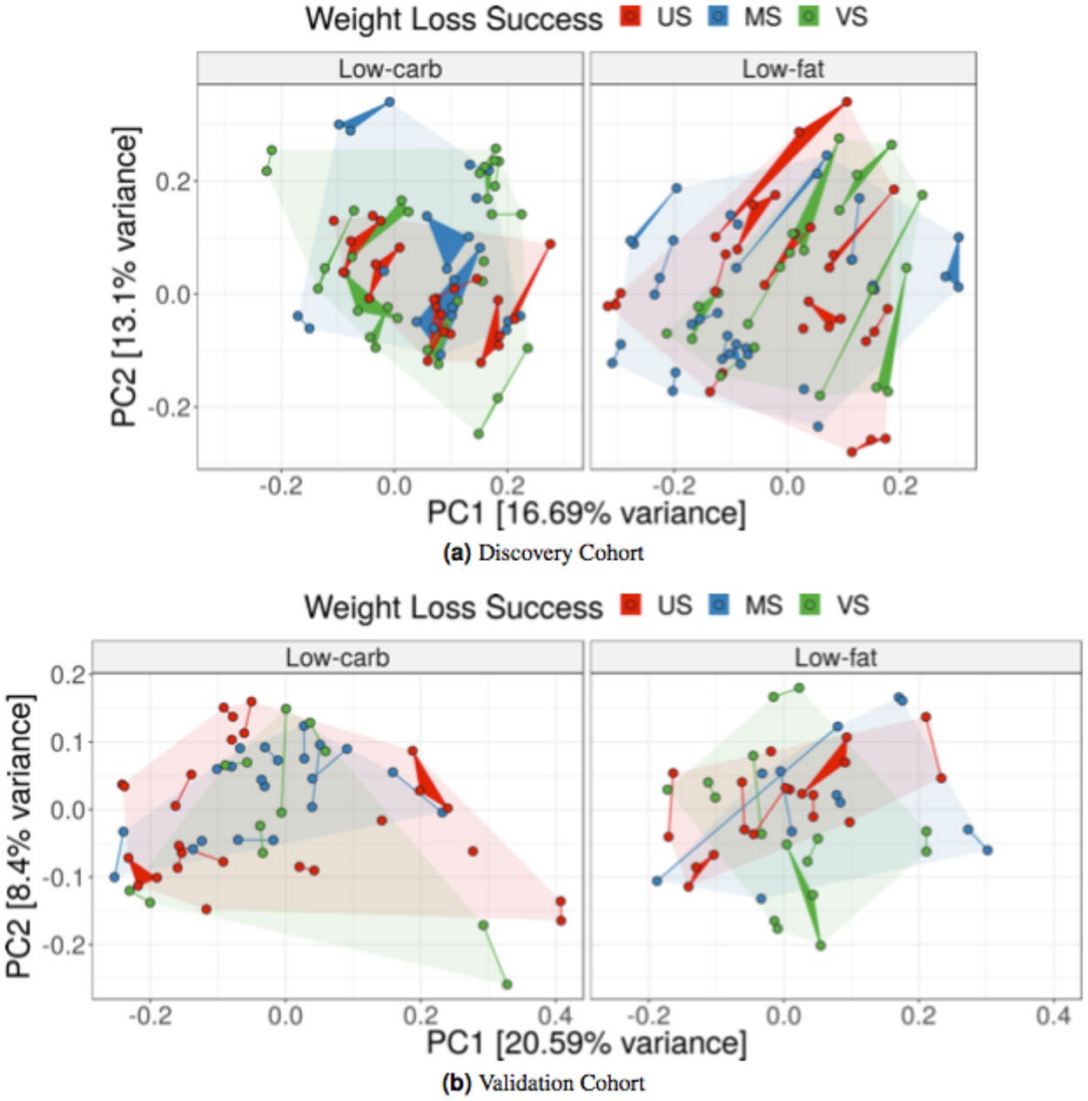
Pre-diet microbial community composition not correlated with weight loss success. Pre-diet fecal microbiota collected from subjects in the (**a**) discovery and (**b**) validation cohorts prior to a low-carb (*left*) or low-fat (*right*) dietary intervention. Each point represents a single fecal sample and samples corresponding to the same subject are connected forming edges or triangles. Colors indicate 12-month weight loss success: very successful (VS), >10% weight loss; moderately successful (MS), 3–10% weight loss; and unsuccessful (US), <3% weight loss. The faded background polygons show convex hulls for corresponding success categories. Principal coordinates analysis was computed with Bray-Curtis distance on inverse-hyperbolic-sine transformed counts.

Pre-diet gut microbial community composition varied among subjects, and samples collected from the same individual tended to cluster together in principal coordinates analysis (Fig. 1). In the discovery cohort, pre-diet microbiota composition did not cluster by 12-month weight loss success category (PERMANOVA on Bray-Curtis dissimilarity low-carb: *p* = 0.51; low-fat: *p* = 0.81). The microbiota composition also did not cluster by age, gender, pre-diet weight, body mass index or dietary adherence in the principal coordinates analysis. Similar results were found in the validation cohort (Fig. 1b).

### Higher gut microbiota plasticity correlated with successful 12-month weight loss

**Figure 2:**
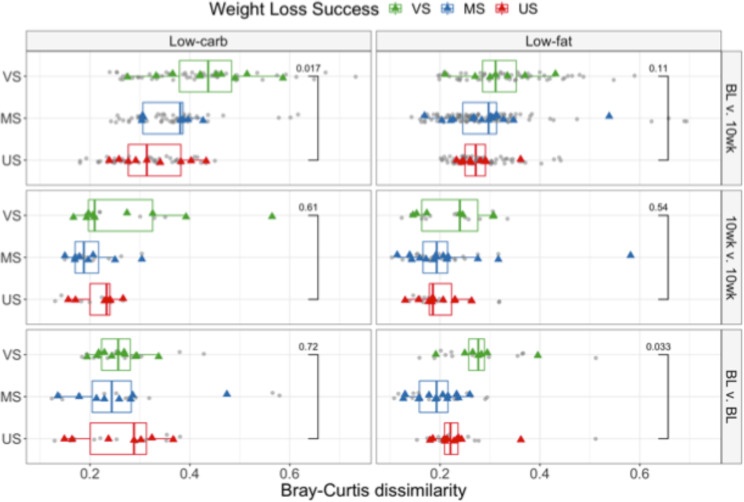
Gut microbiota plasticity over different periods. Pairwise *β*-diversity is shown between daily pre-diet samples (BL v. BL), between daily samples taken 10 weeks after initiation of the dietary intervention (10wk v. 10wk), and between BL and 10-week samples (BL v. 10wk). Bray-Curtis dissimilarities are shown for low-carb (left) and low-fat (right) diets. Grey points indicate computed pairwise dissimilarities between samples; colored points correspond to the average dissimilarity for each subject and are colored by weight loss category: US – Unsuccessful, <3% weight loss; MS – Moderately successful, 3–10% weight loss; VS – Very successful, >10% weight loss. Results with other *β*-diversity metrics are shown in Supplementary Figs. S2 and S3. The Wilcoxon rank sum test was performed to compare the mean difference in plasticity between US and VS groups.

In the discovery cohort, 85% of subjects (n = 26 for low-carb, n = 30 for low-fat) provided two or three fecal samples on consecutive days prior to the start of the intervention. These samples allowed us to quantify subjects’ *pre-diet daily microbiota plasticity*, i.e., the amount of daily variability in an individual’s microbiota composition, measured with *β*-diversity metrics. Pre-diet microbiota plasticity was significantly higher for VS subjects on the low-fat diet compared to US subjects (baseline BL v. BL plots in Fig. 2, Wilcoxon rank-sum test *p* = 0.033 for Bray-Curtis dissimilarity). This trend was present regardless of distance metric (Supplementary Fig. S2a) but was not significant for phylogenetically-aware metrics or when phylogenetically related ASVs were clustered (Supplementary Fig. S2b). There was no difference in pre-diet plasticity between weight loss success groups for the low-carb diet. Across both diet groups, pre-diet daily plasticity was not associated with subject’s food pickiness (Spearman’s rank correlation − 0.10, *p* = 0.45), food neophobia (Spearman’s rank correlation − 0.17, *p* = 0.21) nor with education level (a proxy for socioeconomic status, Spearman’s rank correlation − 0.069, *p* = 0.61). Pre-diet daily plasticity was also not associated with subject’s reported baseline fiber (Spearman’s rank correlation − 0.12, *p* = 0.38), protein (Spearman’s rank correlation 0.065, *p* = 0.64), fat (Spearman’s rank correlation 0.26, *p* = 0.054) or carbohydrate (Spearman’s rank correlation − 0.26, *p* = 0.054) consumption.

Consecutive daily samples were also collected after subjects had been on the dietary intervention for approximately 10 weeks; there was no difference in daily plasticity between weight loss groups at 10 weeks for either diet (10wk v. 10wk plots in Fig. 2, Wilcoxon rank-sum test on Bray-Curtis dissimilarity *p* = 0.61 for low-carb, *p* = 0.54 for low-fat).

We also quantified *plasticity over ten weeks* in response to the dietary intervention, i.e., the variability between gut microbiota composition before the start of the dietary intervention and after 10 weeks of dieting. The plasticity over ten weeks was computed for subjects (n = 28 low-carb, n = 32 low-fat) who provided at least one pre-diet sample and another ten weeks after the start of the dietary intervention (77 ± 9 days apart for low-carb diet, 81 ± 14 days for low-fat). For each subject, the average pairwise *β*-diversity (between each pre-diet and 10-week sample) was calculated. On both diets, VS subjects had higher plasticity between their baseline and 10-week fecal microbiota communities than US subjects (BL v. 10wk plots in Fig. 2; Wilcoxon rank-sum test on Bray-Curtis dissimilarity: low-carb *p* = 0.017; low-fat *p* = 0.11). Again, for both diets the observed trends were similar across multiple *β*-diversity metrics with insignificant differences for phylogenetically-aware metrics. In this analysis, ASV-clustered data closely matched the unclustered results (Supplementary Fig. S3). Daily pre-diet plasticity was positively correlated with plasticity over ten weeks for subjects on the low-fat diet (Spearman’s rank correlation 0.37 for Bray-Curtis, p = 0.053). The magnitude of variation between the pre-diet and the 10-week period was higher than day-to-day plasticity at either the baseline or 10-week time point (Fig. 2, Wilcoxon rank-sum test *p* < 0.0001 for both low-carb and low-fat). Although intra-individual plasticity over ten weeks was significantly higher for VS compared to US subjects, inter-individual differences across subjects were still larger in magnitude on both diets (Supplementary Fig. S4).

Within each cohort, pre-diet phylogenetic *α*-diversity was not different between US and VS subjects (Supplementary Fig. S5) and was not correlated with dietary adherence on either diet. However, for subjects in the discovery cohort, we found a negative correlation between pre-diet plasticity and average *α*-diversity (mean of all pre-diet samples) for subjects on the low-fat diet (Supplementary Fig. S6; Spearman’s rank correlation coefficient − 0.42 for Bray-Curtis dissimilarity, p = 0.021). This suggests that individuals on the low-fat diet with low mean bacterial alpha-diversity tend to exhibit a more variable microbiota composition.

The magnitude of gut microbiota plasticity (using any *β*-diversity metric) was not statistically different between women and men at any time point: pre-diet (Wilcoxon rank-sum test on Bray-Curtis distance *p* = 0.86), at 10-week (*p* = 0.35), or between pre-diet and 10-week (*p* = 0.54). Subjects in the validation cohort did not collect pre-diet fecal samples on consecutive days (median 12 [IQR 7,25] days between samples) and fecal samples from 10-weeks into the intervention were not available, so we were unable to validate plasticity as a factor in weight loss success.

### Gut microbiota plasticity correlated with dietary change in a sex- and diet-dependent manner

**Figure 3:**
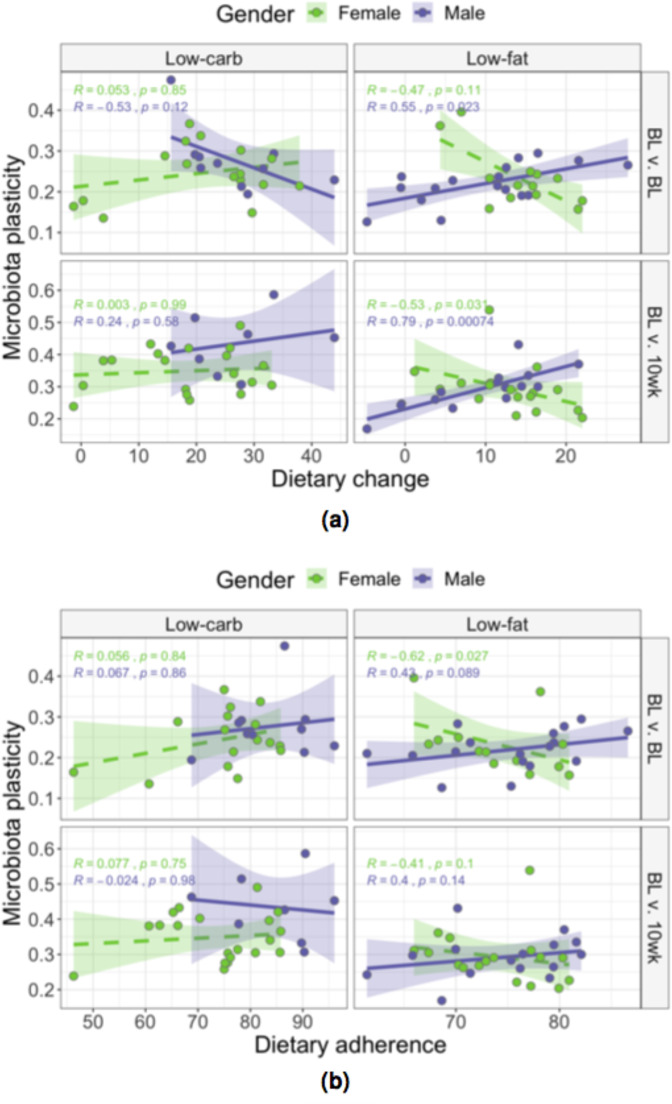
Gut microbiota plasticity correlated with dietary change in a sex- and diet-dependent manner. Spearman’s rank correlations between (**a**) dietary change or (**b**) dietary adherence and plasticity (measured as Bray-Curtis dissimiliarity) between daily pre-diet samples (BL v. BL) and between pre-diet and 10-week samples (BL v. 10wk) are shown for low-carb (left) and low-fat (right) diets. Male (purple) and female (green) subjects show opposite correlations in many cases.

Given sex-specific differences in the larger DIETFITS study population between reported dietary adherence and weight loss, we present sub-group analyses. Women and men on the low-fat diet exhibited different correlations between dietary change and pre-diet daily plasticity (Fig. 3, BL v. BL panel). Men with higher plasticity reduced their fat consumption more (had higher change) than men with lower plasticity (Spearman’s rank correlation using Bray-Curtis dissimilarity 0.55, *p* = 0.02), whereas women lacked a meaningful correlation between plasticity and dietary change (Spearman’s rank correlation − 0.47, *p* = 0.11). For both women and men on the low-carb diet, no significant correlations were observed between dietary change and daily pre-diet plasticity. We suspected that dietary change would affect diet-induced microbiota plasticity over ten weeks; more specifically, we hypothesized that the gut bacterial community composition would shift more in subjects with more drastic changes to their diet while on the intervention. We observed highly significant and opposite correlations between men and women on the low-fat diet (see Fig. 3, BL v. 10wk panel). This was not the case for subjects on the low-carb diet, where again no correlations were noted between diet-induced plasticity and dietary change for both women and men. Trends were consistent across a variety of distance metrics, but failed to reach statistical significance with phylogenetically-aware metrics (Supplementary Fig. S7). The only significant correlation between dietary adherence and plasticity was seen for pre-diet daily plasticity and only for women on the low-fat diet (Spearman’s rank correlation − 0.62, *p* = 0.03 (Fig. 3b).

### Pre-diet *Prevotella*/*Bacteroides* (P/B) ratio correlated with 12-month weight loss success on low-carb diet in discovery, but not validation, cohort

**Figure 4:**
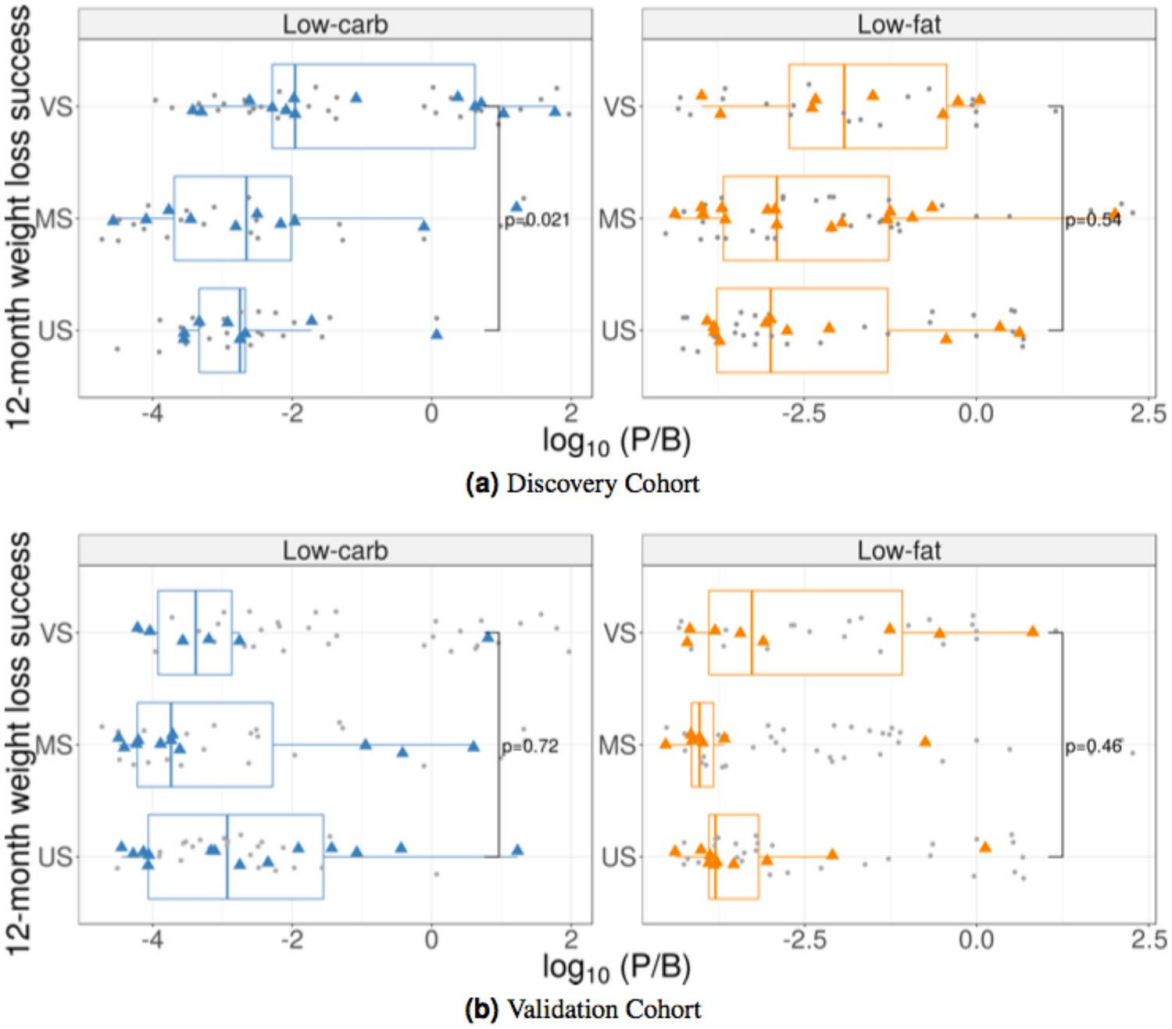
Differences in *Prevotella*/*Bacteroides* (P/B) ratio among weight loss success groups. Subjects on the low-carb diet are shown in the left panels; those on the low-fat diet are shown in the right panels. Grey points indicate P/B ratio for individual samples; colored points correspond to the average P/B ratio for each subject. Data from the (**a**) discovery and (**b**) validation cohort are displayed by subject’s weight loss success category at 12 months after the start of the dietary intervention: US – Unsuccessful; MS – Moderately successful; VS – Very successful. P-values shown for Wilcoxon rank-sum test comparing US and VS groups.

Nearly 50% of subjects had no *Prevotella* sp. detected in their fecal samples and a pseudocount of 1 was substituted for *Prevotella* in the P/B ratio calculation (higher sequencing depth might have resulted in rare *Prevotella* sp. detection). In the discovery cohort, subjects on the low-carb diet with VS weight loss had significantly higher P/B ratio (median = 0.014) compared to US subjects (median = 0.0004; Wilcoxon rank-sum test *p* = 0.021); however, the same was not observed in the validation cohort (VS median P/B ratio = 0.0003; US median = 0.0009; *p* = 0.718; Fig. 4). There was no difference in P/B ratio between US and VS subjects randomized to the low-fat diet for either cohort (discovery: *p* = 0.54; validation: *p* = 0.46).

For a direct comparison of the results presented by Hjorth *et al*.^17^, we classified subjects as high or low P/B ratio (due to the lower prevalence and quantity of *Prevotella* sp., we used a P/B ratio cutoff value of 0.003, instead of 0.01, to distinguish high from low P/B ratio). Subjects on the low-carb diet with high P/B ratio had more weight loss than those with low P/B ratio in the discovery cohort (Wilcoxon rank sum test *p* = 0.003), but not in the validation cohort (Wilcoxon rank sum test *p* = 0.052). Again, there was no association found between weight loss and P/B ratio for subjects on the low-fat diet in either cohort (Wilcoxon rank sum test, discovery *p* = 0.27; validation *p* = 0.55). There was no correlation between the amount of dietary fiber consumed and weight loss for subjects with high P/B ratio, regardless of assigned diet (Spearman rank correlation −0.084, *p* = 0.54 for discovery cohort; 0.15, *p* = 0.59 for validation cohort).

### Differential abundance of sequence clusters not consistently predictive of 12-month weight loss success

**Figure 5:**
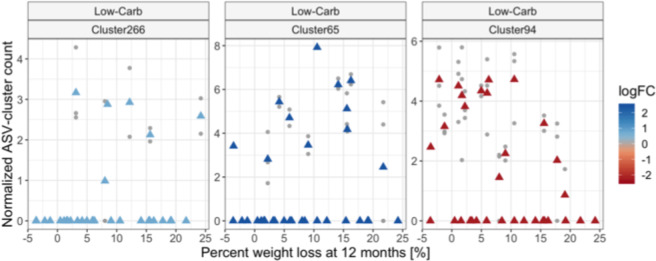
Taxa differentially abundant between weight loss success groups. ASV-clusters found differentially abundant when comparing subjects that were VS compared to US at 12-month weight loss on the low-carb diet. ASV-clusters were normalized and inverse-hyperbolic-sine-transformed for variance stabilization prior to analysis; the normalized, transformed values are shown on the y-axis. ASV-clusters have a median 96.8% sequence similarity (a taxonomic description can be found in Table 2). No taxa were found differentially abundant on the low-fat diet. Grey points represent individual samples and triangles represent the mean value for each subject. Triangle color represents the average log2-fold change (logFC) between subjects in the US vs VS groups; positive values indicate higher counts in VS subjects and negative values indicate higher counts in US subjects.

To evaluate patterns of specific members of the pre-diet microbiota that might predict weight loss success, we tested for clusters of phylogenetically related ASVs that differed in abundance between dieters who achieved VS 12-month weight loss compared to US. We compared within each diet separately, filtering out clusters that were not present in at least 10% of subjects from the focal diet. In the discovery cohort we found one cluster containing 10 Ruminococcaceae ASVs (Cluster94) that was significantly more abundant in US compared to VS subjects on the low-carb diet (*p* = 0.0006) (see Table 2). In contrast, VS subjects had significantly higher abundances of a cluster containing 64 different Ruminococcaceae ASVs (Cluster65, *p* = 0.023)) and a cluster containing 2 *Enterorhabdus* ASVs (Cluster266, *p* = 0.025) compared to US subjects on the low-carb diet. However, the abundances do not display a proportional linear dose-dependent relationship with percentage weight loss for all subjects (Fig. 5), suggesting that these clusters are unlikely to be strong predictors of weight loss success in a larger population. No clusters were identified as differentially abundant for the low-fat diet. Similar results were obtained when differential abundance analysis was performed on the unclustered ASV counts and when a continuous predictor corresponding to the percentage weight loss (instead of categorical: VS vs US) was applied (Supplementary Fig. S8). ASVs and ASV-clusters identified as differentially abundant in the discovery cohort were not significant in the validation cohort (Table 2). In most cases, both the magnitude and direction of effect were discordant between cohorts.

**Table 2:** Log-fold changes of pre-diet ASVs and ASV-clusters found differentially abundant when modeled against weight loss success category at 12 months for low-carb dieters. Values represent log-fold changes between the US and VS groups. Bold values were significant for the cohort indicated. No clusters or ASVs were found differentially abundant in either cohort for the low-fat diet. ^*^p < 0.05, ^**^p < 0.001.

ASV level	Cluster/Seq	Organisms Included	No. Seqs	Cohort	
				Discovery	Validation
ASV-clusters	Cluster94	Ruminococcaceae_UCG-013 sp. & Clostridium_III sp.	10	−2.24**	0.35
	Cluster266	Enterorhabdus sp.	2	0.74*	−0.12
	Cluster65	Ruminococcaceae_UCG-014 sp.	69	2.45*	−1.81
ASVs	Seq175	Ruminococcaceae_UCG-013 sp.	1	−2.19*	−0.31
	Seq64	Bacteroides sp.	1	−1.71*	0.08
	Seq77	Bifidobacterium sp.	1	−560.59	−2.30*
	Seq169	Ruminococcus2 sp.	1	−0.25	−1.78*
	Seq90	Faecalibacterium prausnitzii	1	0.48	2.78*
	Seq746	Enterorhabdus sp.	1	0.88*	−0.15
	Seq182	Ruminococcaceae_UCG-002 sp.	1	1.30*	−0.63

## Discussion

Our study suggests the potential importance of gut microbiota plasticity in sustained weight loss. We have defined microbiota plasticity as variability in the structure and composition of the microbiota (measured by *β*-diversity metrics), over time scales as short as a day. In several earlier studies, individual temporal variability of the gut microbiota was seemingly eclipsed by larger-scale variability across body habitats^23^, severe perturbations^24^, or geographically distinct populations^25^, which may have led some to underestimate the importance of plasticity within an individual. However, a study of weekly samples from 85 adults showed that temporal variability is a personalized feature^26^; with some individuals displaying consistently high or low compositional variability. Our results are the first to illustrate that this personalized feature of the microbiota might be relevant to weight loss.

Higher pre-diet daily plasticity in gut community composition was observed in subjects attaining higher 12-month weight loss, but only on the low-fat diet. This trend was consistent across several *β*-diversity metrics indicating that microbiota was more plastic both in terms of membership and structure, but lost significance when evaluated with phylogenetically-aware distance metrics, and when grouping taxa into phylogenetically-related ASV clusters, implying that closely related species might be replacing each other in subjects with higher daily plasticity. Of note, no pre-diet microbes were differentially abundant for US compared to VS subjects on the low-fat diet, a result that might have been influenced by the increased plasticity found in the latter. In agreement with other studies, we saw a negative correlation with compositional variability and phylogenetic diversity (more diverse communities were less variable)^26–28^. The insurance hypothesis in ecological theory suggests that biologically diverse communities are more resilient as they contain a larger set of community traits/functions that enable them to adapt to changing environments and buffer the system against the loss of species^29^. Here, higher turnover of phylogenetically related microbes might allow for a greater response to the dietary intervention.

As this was seen only for low-fat dieters, we hypothesize that increased plasticity might have facilitated responsiveness to the increased carbohydrates and fiber consumed on the low-fat diet, making the transition to the new diet easier for subjects in ways that aided adherence through appetite suppression^10,30–34^. Our data suggest this is true for men, as we saw positive correlations between plasticity and both weight loss and dietary adherence. Despite magnitudes comparable to men’s daily pre-diet plasticity, women on the low-fat diet displayed a negative trend between plasticity and dietary adherence. This suggests that the mechanisms underlying the relationship between plasticity and weight loss might be independent of dietary adherence for this sub-population. Sex hormones play integral roles in appetite regulation^35,36^. Although we did not measure hormones directly, it is possible that these signals might be differentially stronger than other anorexigenic signals originating from the gut for women compared to men, possibly explaining some of the sexual dimorphism in our results. Another possibility is that self-reporting biases might have influenced the 24-hr dietary recalls, and consequently also affected our dietary adherence measures, differently for men and women^37^. We accounted for this by calculating macronutrient intake as a percentage of total energy intake, but these biases were not assessed directly in the survey instrument and thus we were unable to specifically adjust for them.

Plasticity of the gut microbiota over the first 10 weeks of the dietary intervention was higher for VS compared to US subjects on either diet, but significant only for low-carb dieters. This may be a result of the larger dietary alterations noted for low-carb dieters who reduced carbohydrate intake from 44.6% down to 17.6% of daily kcals, compared to low-fat dieters who reduced their fat consumption from 36.8% of daily kcals down to 21.4%. Plasticity over the 10-week period, in response to new dietary patterns, was higher than daily plasticity at either time point. This was expected due to autocorrelation and also because dietary changes can have strong influences on taxonomic composition and functional capability of the gut microbial community^6,38–40^. However, the non-standardized, self-titrated interventions did not lead to a convergence of subjects to similar gut microbiota communities. After 10 weeks on the dietary intervention, subjects’ community membership and structure were still more similar to their own pre-diet communities rather than to other subjects on the same diet. The repeated measures at both time points (pre-diet and 10 weeks) enabled us to observe that the plasticity of the microbial community in response to the diet was higher than daily variability at either time point.

A limitation of our study is that daily microbiota plasticity could be influenced by lifestyle and sample-specific factors that we did not measure, such as diversity of dietary intake, number of people living in the household, presence of pets in the household and time of day of sample collection^41–43^. Our study design does not allow us to make any causal inference on the relationship, and we do not have complimentary data (e.g. metabolite plasticity) to interrogate potential mechanisms of action by which microbiota variability might be exerting influence on adherence or weight loss. Furthermore, we were unable to evaluate the robustness of these findings in our validation cohort due to differences in sampling regimes. Therefore, the correlation between microbiota plasticity and successful weight loss should be corroborated in future cohorts, potentially with more frequent sample collection to establish the plasticity as a distinctly personalized feature and not influenced by sample-specific properties.

Our study highlighted the importance of validation cohorts in microbiota studies with small to moderate sample sizes. Contrary to our hypothesis, we found no specific taxonomy-related microbial features clearly associated with 12-month weight loss success across both cohorts. This could be due to our small sample size (n=25-34 for any diet-cohort combination), indeed, only ~ 20% of ASV-clusters were detected in at least half of the subjects on either diet, limiting our statistical power to identify differences between VS and US subjects. Our sample size was comparable to other published studies investigating the gut microbiota and diet responsiveness^17,18,44,45^, however we included stringent filtering of samples with at least 10,000 reads and ASVs or ASV-clusters present in at least 10% of subjects on the focal diet. We also included replicate samples for most subjects in order to reduce false positive identification during differential abundance testing. The majority of pre-diet ASVs and ASV-clusters identified as differentially abundant between weight loss groups on the low-carb diet were discordant across the discovery and validation cohorts in both magnitude and direction. In addition, previous results that subjects with higher pre-diet P/B ratio had higher 12-month weight loss was corroborated in the discovery cohort, but only for subjects on the low-carb diet and the finding was not confirmed in the validation cohort. These inconsistencies could be the result of the significantly lower dietary adherence and weight loss achieved by low-carb dieters in the validation cohort compared to subjects from the discovery cohort, although fiber consumption was not different across weight loss groups and was similar to the previous studies. The inclusion of a validation cohort illustrated the challenges in generalizing findings from an initial modest sample size to even a similar population. Future research, specifically designed and powered for the outcome of interest, will be necessary to determine if specific taxa or higher P/B ratio are important for long-term weight loss in larger populations.

Gut microbiota plasticity has not been previously studied in relation to sustained weight loss or dietary adherence. Here we present data that suggests the plasticity of the gut microbiota may be related to both in a sex- and diet-specific manner. Our work highlights the importance of investigating kinetic trends in gut bacterial community composition, including long-term shifts and daily plasticity, in addition to explicitly seeking static microbiota signatures and patterns, when studying individual differences and predisposition to successful weight loss.

## Methods

This research was approved by the Panel on Human Subjects in Medical Research (protocol 22035) at Stanford University, Stanford, CA, USA, and all procedures were in accordance with the Helsinki Declaration of 1975 as revised in 1983. All participants provided written informed consent.

### Study population and sample collection

Overweight and obese adults in the Diet Intervention Examining The Factors Interacting with Treatment Success (DIETFITS) study enrolled between Fall 2013 and Spring 2014 were approached for inclusion in this study. DIETFITS was a 12-month randomized clinical trial of low-carb and low-fat diets^19,20^ (clinical trial registration NCT01826591). The diets had no specific caloric, fat or carbohydrate restrictions, but instead involved counseling sessions focused on three main components central to sustaining a low-carb or low-fat diet. First, for the initial eight weeks, subjects were instructed to progressively reduce either carbohydrate or fat intake as much as possible (with an objective of achieving 20 g/day), and maintain their lowest possible intake for at least several weeks. Second, after the initial eight weeks they were then encouraged to titrate their intake by increasing fat or carbohydrate consumption by increments of 5-15 g/day and maintaining that intake for a week or more while noting their satisfaction (e.g., satiety, palatability) and weight loss success. After settling on a sustainable target (one that left them feeling full but still able to lose weight), they were asked to maintain that intake level for the remainder of the study. The third component of the study’s strategy was promotion of a high-quality diet focused on whole, real foods, that are mostly prepared at home and that contain as many vegetables as possible. Subjects were assigned to attend 22 in-person instructional sessions related to nutrition, behavior, emotions, and physical activity. Participant data, including clinical outcomes, was collected and managed using REDCap electronic data capture tools hosted at Stanford University^46^. Subjects from the discovery cohort were asked to provide self-collected fecal samples from three consecutive days at two separate time points: pre-diet and 10 weeks after initiation of the dietary intervention. The 10-week time point was selected because subjects in long-term weight-loss studies typically reach their maximum weight loss around 3 months^47^ and we expected maximum change in the microbiota to co-occur with this peak weight loss. Subjects from the validation cohort were asked to provide two self-collected pre-diet fecal samples. Fecal samples were stored at −20 °C until delivered to the lab and then at −80 °C until processing. A total of 424 fecal samples with sufficient sequencing depth in high-quality reads (>10,000) were collected from 66 discovery cohort and 56 validation cohort subjects who provided complete weight data.

### Dietary adherence

Three (two weekday and one weekend) unannounced 24-hour dietary recalls were conducted for each participant at baseline (prior to diet assignment) and again at approximately 3, 6, and 12 months of dietary intervention. On average, each diet resulted in a reduced intake of the restricted component (fats or carbs) but also in the number of total calories consumed, so we first determined dietary adherence as the percentage of total kcals coming from non-restricted foods (an average was taken over the dietary recalls taken at 3, 6, and 12 months). Higher dietary adherence values indicate a larger percentage of the reported dietary intake did not include foods the subject specifically set to restrict. We also calculated diet change, the difference in consumption of restricted foods between baseline (pre-diet) and during the dietary intervention (the average from 3, 6, and 12 months). Dietary change was measured as decrease in percentage of total daily kcals consumed from restricted foods (fats/carbs) and acts as a proxy for how much a subject’s dietary practices changed on the intervention. The dietary change value was included as it might have greater influence on the microbiota than adherence alone, depending on an individual’s pre-intervention dietary practices. For example, a subject who typically consumed 20% of their diet as fats prior to the intervention and reduced their fat intake to 10% on the low-fat diet would have a dietary adherence of 90% but change of only 10%. Whereas a subject with a pre-diet fat consumption of 35% who reduced it to 10% would also have a dietary adherence of 90% but a dietary change of 25%.

### DNA extraction and 16S rRNA gene sequencing

DNA was extracted from 50-150mg fecal material using the Qiagen PowerSoil DNA Isolation Kit (Qiagen, Venlo, The Netherlands) with the following protocol modifications: samples were incubated in lysis buffer at 65 °C for 10 minutes, bead beating was conducted for 20 minutes, and all subsequent vortexing steps were replaced with gentle but thorough inversions. Extracted DNA was stored at −20 °C. The V4 region of the 16S rRNA gene was amplified using the protocol of Caporaso *et al*.^48^. Briefly, samples were amplified in triplicate 25ul PCRs in 96-well plates with the final volumes per reaction: water 10.9 ul, MasterMix (5 PRIME HotMasterMix) 10 ul, reverse primer 0.1 ul, forward primer 1 ul, template DNA 3 ul. Replicates were run simultaneously on 3 thermal cyclers for 30 cycles of: 94 °C for 45 s, 52 °C for 60 s, 72 °C for 90 s, with a ten minute extension at 72 °C at the end. Amplification was verified by gel electrophoresis on pooled replicates (failures were repeated) and bands were excised and cleaned (MO BIO UltraClean-htp 96 Well PCR Clean-Up Kit) per manufacturer protocol. DNA was quantified (Invitrogen Quant-iT dsDNA Assay Kit, High Sensitivity) using a microplate reader (FLEXstation II 384-Fluorescent 6), and amplicons were combined in equimolar ratios. This pooled DNA was concentrated via ethanol precipitation and then resuspended in nuclease-free water. DNA libraries were submitted to the Functional Genomics Facility at Stanford University for sequencing on an Illumina MiSeq (2 × 250 bp) over 7 separate runs, yielding 73.3M raw reads.

### DADA2 amplicon sequence variant (ASV) sample inference and tree building

The DADA2 R package^49^ was used for quality filtering, denoising, chimera removal and sequence inference (obtaining sequence counts). Forward reads were trimmed to length 240 in all but one sequencing run (length of 230 due to lower quality); reversed reads, commonly with lower quality, were trimmed to length ranging from 160-200 across sequencing runs. After merging, default settings were used for error estimation and denoising with the following exception: maxEE = 2, meaning that merged reads with expected error higher than maxEE were discarded (where *EE* = ∑10^−*Q*∕10^). Finally, after amplicon count inference, chimeras were removed and sequences of length 230-234 bp were retained; 37.5M (51%) reads passed filtering criteria. Taxonomic assignment of the 4,234 unique sequences was performed according to the Bioconductor workflow^49,50^ using RDP trainset 16^51^ and Silva v128^52^ databases.

#### Phylogenetic tree estimation

We estimated a phylogenetic tree from obtained sequences in accordance with the Bioconductor workflow^50^. First, we performed multiple alignment using the DECIPHER R package^53^, and then used phangorn^54^ to fit a Generalized time-reversible Gamma rate variation (GTR+G+I) maximum likelihood tree initialized at the neighbor-joining tree (parameters *k*=4 and inv=0.2 were used for phangorn::pml function).

#### Sequence clustering using phylogeny

Due to high resolution of the DADA2 pipeline, the obtained amplicon sequence variant (ASV) count-matrix was very sparse. In order to attain more overlap in organism counts between samples and subjects, we clustered ASVs into phylogenetically similar sequence groups using the estimated phylogenetic tree. These clusters were generated by cutting the associated phylogenetic tree at the height, *h* = 0.1, corresponding to median difference between member sequences equal to 7.5 bp (out of 233 bp). This is equivalent to 96.8% sequence identity, which correlates with thresholds to differentiate genera^55,56^. We used both the original ASV data and ASV-clusters for downstream analysis. This ASV-clustering approach was preferred over the traditional OTU clustering: ASVs reduce false positive rates more than most OTU clustering approaches and therefore result in a more accurate phylogenetic tree and more accurate, tractable clusters^57–60^.

### Statistical analyses

R scripts used for the analysis are available in Supplementary Files 2–10.

#### Ordination

To visualize the data we used principal coordinates analysis with Bray-Curtis distance applied to inverse-hyperbolic-sine-transformed count data. The transformation prevents the Bray-Curtis dissimilarity metric from placing too much weight on species/ASVs highly abundant in all samples. All samples were included together in the computation of the ordination projection. The plots were then faceted into distinct dietary intervention assignments. Differences in community composition were tested using PERMANOVA (adonis function from the vegan package^61^, permutation = 999).

#### Microbiota diversity and plasticity

Gut microbiota phylogenetic *α*-diversity was estimated using the procedure of Nippress *et al*.^62^, based on rarefaction curves. Phylogenetic diversity was evaluated at an interpolated depth (rarefaction size) of 11,000 sequence reads – the minimum library size of samples post-filtering.

Daily pre-diet gut microbiota variability was estimated using pairwise *β*-diversity between each subject’s pre-diet samples. If a subject provided three samples, only the pairwise *β*-diversity for consecutive days were used to compute the average. Consecutive samples in the discovery cohort were taken a median of 1 day apart (IQR 1, 2) with an exception of a few subjects who provided samples up to 5 days apart. The time span between an individual subject’s samples was balanced across weight loss groups. To estimate variability between gut microbiota pre-diet and 10 weeks into the dietary intervention, we calculated the pairwise *β*-diversity between each pre-diet and 10-week sample for each subject, and then calculated the mean across all pairwise comparisons. For all plasticity measures, Jaccard dissimilarity and unweighted UniFrac distance were used to asses the variability in taxa presence/absence, whereas Bray-Curtis dissimilarity, weighted UniFrac distance, and Jensen-Shannon divergence were used to estimate variability in abundances. The difference in variability between VS and US subjects was tested for significance separately for each diet using non-parametric Wilcoxon rank-sum test.

#### Differential abundance testing

We looked for pre-diet ASV-clusters, and single ASVs, that were differentially abundant with respect to 12-month weight loss. The inclusion of consecutive daily samples (repeated measures) for subjects allowed for a more accurate estimation of the underlying processes and helped reduce false discoveries. The limma differential abundance (DA) testing framework^63^ is the most conservative analysis that allowed us to model variability of repeated measurements as within-subject random effects. Before using limma we transformed the raw count data by first computing the library size factors using estimateSizeFactors from DESeq2 package^64^ with the argument type “poscounts”, which was specifically developed for sparse sequencing data. We used a customized version of limma::voom function, where we substituted the log2-counts per million (logCPM) transformation intended for bulk human RNA-seq data, with an inverse-hyperbolic-sine $({sinh}^{-1}(x)=log(x+\sqrt{1+{x}^{2}}))$ transformation shown to be more appropriate for data which follows a negative binomial distribution (as is the case for 16S rRNA gene sequencing)^65–68^. Functions for differential abundance estimation from limma were then used to fit the pre-diet ASV or ASV-cluster abundances. For increased modeling accuracy and higher power, the method was applied to all samples from both diets together. Testing was performed by evaluating the contrast between weight loss success categories within each diet separately (using diet-weight loss interaction terms in the model design). An additional fixed-effect term for sample sequencing lane was included in the model to account for batch effects from different sequencing runs. For each of the two diets we tested both the contrast between subjects from the VS compared to US weight loss group, and also conducted secondary analyses modeling the outcome as a continuous variable (percent weight lost at 12-months). Only the taxa found in at least 10% of subjects on the given diet and significant after adjusting for multiple hypothesis testing (Benjamini-Hochberg method^69^) at level *α* = 0.05 were retained.

### Accession codes

All sequencing reads are deposited in the Sequence Read Archive under BioProject PRJNA542910.

## Supplementary information

Supplementary Figures and Tables.

## References

[1] Ng M (2014). Global, regional, and national prevalence of overweight and obesity in children and adults during 1980–2013: a systematic analysis for the Global Burden of Disease Study 2013. The Lancet.

[2] Tremmel M (2017). Economic Burden of Obesity: A Systematic Literature Review. International Journal of Environmental Research and Public Health.

[3] Johnston BC (2014). Comparison of weight loss among named diet programs in overweight and obese adults: A meta-analysis. JAMA - Journal of the American Medical Association.

[4] Dansinger ML, Gleason JA, Griffith JL, Selker HP, Schaefer EJ (2005). Comparison of the Atkins, Ornish, Weight Watchers, and Zone Diets for Weight Loss and Heart Disease Risk Reduction. JAMA.

[5] Alhassan, S., Kim, S., Bersamin, A., King, A. C. & Gardner, C. D. Dietary adherence and weight loss success among overweight women: Results from the A to Z weight loss study. *International Journal of Obesity* **32**, 985–991, 10.1038/ijo.2008.8, NIHMS150003 (2008). 10.1038/ijo.2008.8 PMC4005268 18268511

[6] David LA (2013). Diet rapidly and reproducibly alters the human gut microbiome. Nature.

[7] Walker AW (2011). Dominant and diet-responsive groups of bacteria within the human colonic microbiota. The ISME Journal.

[8] Ley R, Turnbaugh P, Klein S, Gordon J (2006). Microbial ecology: human gut microbes associated with obesity. Nature.

[9] Turnbaugh, P. J. *et al*. An obesity-associated gut microbiome with increased capacity for energy harvest. *Nature* **444**, 1027–1031, 10.1038/nature05414, t8jd4qr3m (2006). 10.1038/nature05414 17183312

[10] Holzer, P., Reichmann, F. & Farzi, A. Neuropeptide Y, peptide YY and pancreatic polypeptide in the gut-brain axis, 10.1016/j.npep.2012.08.005 (2012). 10.1016/j.npep.2012.08.005 PMC3516703 22979996

[11] Cani, P. D. & Knauf, C. How gut microbes talk to organs: The role of endocrine and nervous routes, 10.1016/j.molmet.2016.05.011 (2016). 10.1016/j.molmet.2016.05.011 PMC5004142 27617197

[12] Olivares, M. *et al*. The Potential Role of the Dipeptidyl Peptidase-4-Like Activity From the Gut Microbiota on the Host Health. *Frontiers in Microbiology* **9**, 10.3389/fmicb.2018.01900 (2018). 10.3389/fmicb.2018.01900 PMC6113382 30186247

[13] Breton J (2016). Gut commensal E. coli proteins activate host satiety pathways following nutrient-induced bacterial growth. Cell Metabolism.

[14] Lin, H. V. *et al*. Butyrate and propionate protect against diet-induced obesity and regulate gut hormones via free fatty acid receptor 3-independent mechanisms. *PLoS One* **7**, 10.1371/journal.pone.0035240 (2012). 10.1371/journal.pone.0035240 PMC3323649 22506074

[15] Psichas A (2015). The short chain fatty acid propionate stimulates GLP-1 and PYY secretion via free fatty acid receptor 2 in rodents. International Journal of Obesity.

[16] Healey GR, Murphy R, Brough L, Butts CA, Coad J (2017). Interindividual variability in gut microbiota and host response to dietary interventions. Nutrition Reviews.

[17] Hjorth MF (2018). Pre-treatment microbial Prevotella-to-Bacteroides ratio, determines body fat loss success during a 6-month randomized controlled diet intervention. International Journal of Obesity.

[18] Hjorth MF (2019). Prevotella-to-Bacteroides ratio predicts body weight and fat loss success on 24-week diets varying in macronutrient composition and dietary fiber: results from a post-hoc analysis. International Journal of Obesity.

[19] Stanton M (2017). DIETFITS study (diet intervention examining the factors interacting with treatment success) – Study design and methods. Contemporary Clinical Trials.

[20] Gardner CD (2018). Effect of Low-Fat vs Low-Carbohydrate Diet on 12-Month Weight Loss in Overweight Adults and the Association With Genotype Pattern or Insulin Secretion. JAMA.

[21] Wing RR, Phelan S (2005). Long-term weight loss maintenance. Am. J. Clin. Nutr..

[22] Jeffery RW (2000). Long-Term Maintenance of Weight Loss: Current Status. Heal. Psychol..

[23] Costello EK (2009). Bacterial Community Variation in Human Body Habitats AcrossSpace and Time. Science.

[24] Dethlefsen L, Relman DA (2011). Incomplete recovery and individualized responses of the human distal gut microbiota to repeated antibiotic perturbation. Proceedings of the National Academy of Sciences of the United States of America.

[25] Yatsunenko T (2012). Human gut microbiome viewed across age and geography. Nature.

[26] Flores, G. E. *et al*. Temporal variability is a personalized feature of the human microbiome. *Genome biology* **15**, 531, 10.1186/s13059-014-0531-y, NIHMS150003 (2014). 10.1186/s13059-014-0531-y PMC4252997 25517225

[27] Coyte KZ, Schluter J, Foster KR (2015). The ecology of the microbiome: Networks, competition, and stability. Science (New York, N.Y.).

[28] Martínez I (2015). The Gut Microbiota of Rural Papua New Guineans: Composition, Diversity Patterns, and Ecological Processes. Cell Reports.

[29] Yachi S, Loreau M (1999). Biodiversity and ecosystem productivity in a fluctuating environment: The insurance hypothesis. Proceedings of the National Academy of Sciences.

[30] Larraufie P (2018). SCFAs strongly stimulate PYY production in human enteroendocrine cells. Scientific Reports.

[31] Byrne CS, Chambers ES, Morrison DJ, Frost G (2015). The role of short chain fatty acids in appetite regulation and energy homeostasis. International Journal of Obesity.

[32] Gee JM, Johnson IT (2005). Dietary lactitol fermentation increases circulating peptide YY and glucagon-like peptide-1 in rats and humans. Nutrition.

[33] Cani PD (2009). Gut microbiota fermentation of prebiotics increases satietogenic and incretin gut peptide production with consequences for appetite sensation and glucose response after a meal. American Journal of Clinical Nutrition.

[34] Batterham RL (2002). Gut hormone PYY3-36 physiologically inhibits food intake. Nature.

[35] Hirschberg AL (2012). Sex hormones, appetite and eating behaviour in women. Maturitas.

[36] Hirscbberg AL (1998). Hormonal regulation of appetite and food intake. Annals Medicine.

[37] Hebert JR (1997). Gender Differences in Social Desirability and Social Approval Bias in Dietary Self-report. American Journal of Epidemiology.

[38] Faith JJ, Mcnulty NP, Rey FE, Gordon JI (2011). Response to Diet in Gnotobiotic Mice. Science.

[39] Shoaie S (2015). Quantifying diet-induced metabolic changes of the human gut microbiome. Cell Metabolism.

[40] Mcorist AL (2011). Fecal Butyrate Levels Vary Widely among Individuals but Are Usually Increased by a Diet High in Resistant Starch 1,2. J. Nutr.

[41] Azad MB (2013). Infant gut microbiota and the hygiene hypothesis of allergic disease: impact of household pets and siblings on microbiota composition and diversity. Allergy, Asthma, & Clin. Immunol..

[42] Song SJ (2013). Cohabiting family members share microbiota with one another and with their dogs. eLife.

[43] Liang X, Bushman FD, FitzGerald GA (2015). Rhythmicity of the intestinal microbiota is regulated by gender and the host circadian clock. Proc. Natl. Acad. Sci..

[44] Korpela, K. *et al*. Gut microbiota signatures predict host and microbiota responses to dietary interventions in obese individuals. *PLoS One* **9**, 10.1371/journal.pone.0090702 (2014). 10.1371/journal.pone.0090702 PMC3946202 24603757

[45] Dao MC (2016). Akkermansia muciniphila and improved metabolic health during a dietary intervention in obesity: Relationship with gut microbiome richness and ecology. Gut.

[46] Harris PA (2009). Research electronic data capture (REDCap)—A metadata-driven methodology and workflow process for providing translational research informatics support. Journal of Biomedical Informatics.

[47] Hall KD, Kahan S (2018). Maintenance of Lost Weight and Long-Term Management of Obesity. Medical Clinics of North America.

[48] Caporaso JG (2012). Ultra-high-throughput microbial community analysis on the Illumina HiSeq and MiSeq platforms. The ISME J..

[49] Callahan BJ (2016). DADA2: High-resolution sample inference from Illumina amplicon data. Nature Methods.

[50] Callahan, B., Sankaran, K., Fukuyama, J., McMurdie, P. & Holmes, S. Bioconductor workflow for microbiome data analysis: from raw reads to community analyses [version 1; referees: 3 approved]. *F1000Research* **5**, 10.12688/f1000research.8986.1 (2016). 10.12688/f1000research.8986.1 PMC4955027 27508062

[51] Wang, Q., Garrity, G. M., Tiedje, J. M. & Cole, J. R. Naïve Bayesian classifier for rapid assignment of rRNA sequences into the new bacterial taxonomy. *Appl. Environ. Microbiol*. **73**, 5261–5267, 10.1128/AEM.00062-07, Wang,Qiong,2007,Naive (2007). 10.1128/AEM.00062-07 PMC1950982 17586664

[52] Quast C (2013). The silva ribosomal rna gene database project: improved data processing and web-based tools. Nucleic Acids Res..

[53] Wright ES (2016). Using decipher v2.0 to analyze big biological sequence data in r. The R J..

[54] Schliep K (2011). phangorn: phylogenetic analysis in r. Bioinformatics.

[55] Bosshard PP, Abels S, Zbinden R, Böttger EC, Altwegg M (2003). Ribosomal DNA Sequencing for Identification of Aerobic Gram-Positive Rods in the Clinical Laboratory (an 18-Month Evaluation). Journal of Clinical Microbiology.

[56] Větrovský T, Baldrian P (2013). The variability of the 16s rrna gene in bacterial genomes and its consequences for bacterial community analyses. PLoS One.

[57] Callahan BJ, McMurdie PJ, Holmes SP (2017). Exact sequence variants should replace operational taxonomic units in marker-gene data analysis. ISME J..

[58] Nearing, J. T., Douglas, G. M., Comeau, A. M. & Langille, M. G. Denoising the Denoisers: An independent evaluation of microbiome sequence error-correction approaches.PeerJ2018, e5364, 10.7717/peerj.5364 (2018). 10.7717/peerj.5364 PMC6087418 30123705

[59] Caruso, V., Song, X., Asquith, M. & Karstens, L. Performance of Microbiome Sequence Inference Methods in Environments with Varying Biomass. *mSystems* **4**, 10.1128/msystems.00163-18 (2019). 10.1128/mSystems.00163-18 PMC6381225 30801029

[60] Forster, D. *et al*. Improving eDNA-based protist diversity assessments using networks of amplicon sequence variants. *Environ. Microbiol*., 10.1111/1462-2920.14764 (2019). 10.1111/1462-2920.14764 31361938

[61] Oksanen, J. *et al*. vegan: Community Ecology Package, R package version 2.5-3 (2018).

[62] Nipperess DA, Matsen FA (2013). The mean and variance of phylogenetic diversity under rarefaction. Methods Ecol Evol.

[63] Ritchie ME (2015). limma powers differential expression analyses for RNA-sequencing and microarray studies. Nucleic Acids Research.

[64] Love MI, Huber W, Anders S (2014). Moderated estimation of fold change and dispersion for RNA-seq data with DESeq2. Genome Biol..

[65] Laubscher NF (1961). On stabilizing the binomial and negative binomial variances. J. Am. Stat. Assoc..

[66] Hoyle MH (1973). Transformations: An introduction and a bibliography. Int. Stat. Rev./Revue Int. de Stat..

[67] Burbidge JB, Magee L, Robb AL (1988). Alternative transformations to handle extreme values of the dependent variable. J. Am. Stat. Assoc..

[68] Huber W, von Heydebreck A, Sültmann H, Poustka A, Vingron M (2002). Variance stabilization applied to microarray data calibration and to the quantification of differential expression. Bioinformatics.

[69] Benjamini, Y. & Hochberg, Y. Controlling the False Discovery Rate: A Practical and Powerful Approach to Multiple Testing. *J. Royal Stat. Soc. Ser. B (Methodological)* **57**, 289–300, 10.2307/2346101, 95/57289 (1995).

