# Author Correction: Gut microbiota plasticity is correlated with sustained weight loss on a low-carb or low-fat dietary intervention

**DOI:** 10.1038/s41598-020-68280-z

**Published:** 2020-07-01

**Authors:** Jessica A. Grembi, Lan H. Nguyen, Thomas D. Haggerty, Christopher D. Gardner, Susan P. Holmes, Julie Parsonnet

**Affiliations:** 1 Department of Civil and Environmental Engineering, Stanford University, 318 Campus Drive E250 Clark Center, Stanford, CA 94305 USA; 2 Department of Medicine, Stanford University School of Medicine, 291 Campus Drive, Stanford, CA 94305 USA; 3 Institute for Computational and Mathematical Engineering, Stanford University, 475 Via Ortega, Stanford, CA 94305 USA; 4 Stanford Prevention Research Center, Department of Medicine, Stanford University School of Medicine, 1265 Welch Road, Stanford, CA 94305 USA; 5 Department of Statistics, Stanford University, 390 Serra Mall, Stanford, CA 94305 USA; 6 Department of Health Research and Policy, Stanford University School of Medicine, 150 Governor’s Ln, Stanford, CA 94305 USA

Correction to: *Scientific Reports* https://doi.org/10.1038/s41598-020-58000-y, published online 29 January 2020

This Article contains an error in the Statistical Analysis section where,

"R scripts used for the analysis are available in Supplementary Files 2–10."

should read:

"R scripts used for the analysis are available at https://doi.org/10.5281/zenodo.3763407 and https://github.com/jgrembi/DIETFITS_GutMicrobiotaPlasticity"

