# Supplementary material for: Gut microbiota plasticity is correlated with sustained weight loss on a low-carb or low-fat dietary intervention
Source: Sci Rep. 2020 Jan 29;10:1405. doi: 10.1038/s41598-020-58000-y (PMC6989501; doi:10.1038/s41598-020-58000-y)
Supplement: Supplementary file 1 — Supplementary Figures and Tables. [file 41598_2020_58000_MOESM1_ESM.pdf]

Gut microbiota plasticity is correlated with sustained weight loss on a low-carb or low-fat dietary intervention

Jessica A Grembi[a,†,*], Lan H Nguyen[b,†], Thomas D Haggerty[c], Christopher D Gardner[d], Susan P Holmes[e], and Julie Parsonnet[c,f]

[a] *Department of Civil and Environmental Engineering, Stanford University, 318 Campus Drive E250 Clark Center, Stanford, CA 94305*
[b] *Institute for Computational and Mathematical Engineering, Stanford University, 475 Via Ortega, Stanford, CA 94305*
[c] *Department of Medicine, Stanford University School of Medicine, 291 Campus Drive, Stanford, CA 94305*
[d] *Stanford Prevention Research Center, Department of Medicine, Stanford University School of Medicine, 1265 Welch Road, Stanford, CA 94305*
[e] *Department of Statistics, Stanford University, 390 Serra Mall, Stanford, CA 94305*
[f] *Department of Health Research and Policy, Stanford University School of Medicine, 150 Governor's Ln, Stanford, CA 94305*
[†] *These authors contributed equally to this work*
[*] *Corresponding Author: jgrembi@stanford.edu*

Supplementary Table S1: Dietary consumption and adherence measures by weight loss group, diet, and cohort.

	Low-carb						Low-fat					
	Discovery			Validation			Discovery			Validation		
	US	MS	VS	US	MS	VS	US	MS	VS	US	MS	VS
**Sex, n (%)**												
female	9 (100)	6 (60)	7 (53.8)	11 (78.6)	11 (100)	3 (50)	6 (54.5)	8 (53.3)	3 (37.5)	8 (80)	5 (71.4)	6 (75)
**Fat, % kcal (SD)**												
Baseline	37.6 (3.4)	41.9 (7.1)	37.4 (6.2)	37.5 (6.5)	36.4 (7.6)	38.4 (4.3)	34.5 (8)	36.9 (5.9)	39.6 (4.3)	37.3 (5.5)	37.6 (5.5)	33.2 (3.8)
3 months	55.1 (12.9)	58.9 (7.6)	56.8 (7.8)	44.7 (8)	48.1 (6.5)	57 (8.9)	23.1 (7.9)	22.3 (5.7)	17.6 (8.4)	28 (5.4)	25.7 (8.1)	20.4 (7.7)
6 months	46.8 (9.7)	56.4 (8.9)	55.8 (7.1)	41.5 (6.6)	45 (5.8)	52.2 (7.7)	26.7 (9.2)	25.4 (7.6)	24.9 (6.1)	32.4 (7.5)	26.5 (7.2)	22.4 (10.3)
12 months	41 (12)	47.1 (8.8)	51.9 (7.5)	41.1 (7.8)	41.9 (7.3)	51.7 (8)	29 (7.3)	29.3 (7.5)	27 (11.2)	34.3 (4.2)	35.7 (8.6)	24.3 (6.8)
**Carbohydrates, % kcal (SD)**												
Baseline	46.9 (4.4)	39.7 (7.7)	46.7 (8)	46.4 (5.9)	47.2 (7.7)	42.4 (5.6)	49.8 (8.3)	45.9 (6.3)	42.6 (4.6)	44.5 (6.6)	44.6 (6.1)	51.7 (4)
3 months	24.1 (16.2)	15.2 (7.8)	15.4 (8.2)	32.4 (10.4)	27.6 (8.9)	17 (5)	58.5 (10.8)	57.3 (4.9)	63 (12.3)	50.9 (7.7)	54 (10.9)	59.2 (8.5)
6 months	30.4 (11.7)	19.1 (11.2)	19.1 (10.3)	35.4 (11.2)	31.2 (7.2)	23.2 (10.1)	55.2 (8.1)	55.9 (7.7)	55.6 (9.2)	47.2 (8)	55.7 (9.1)	56.3 (10.1)
12 months	34 (13.3)	28.7 (11.9)	22.1 (10.9)	38.7 (9.6)	35.9 (9.2)	25.5 (8.9)	53.8 (8.2)	52.4 (6.7)	54.3 (11.1)	45.1 (6.1)	43.6 (8.5)	55.3 (7.2)
**Protein, % kcal (SD)**												
Baseline	15.5 (3)	18.4 (4.1)	16 (2.6)	16.1 (2.7)	16.4 (2.9)	19.2 (2.4)	15.7 (3)	17.2 (3)	17.8 (4.4)	18.2 (2.8)	17.8 (4)	15.1 (2.8)
3 months	20.8 (4.4)	25.9 (3.7)	27.8 (6.8)	22.9 (4.9)	24.3 (7.3)	26 (6.8)	18.4 (4.4)	20.4 (4.3)	19.4 (5.6)	21.1 (4.4)	20.3 (8.7)	20.4 (3.1)
6 months	22.8 (5.6)	24.5 (5.2)	25.1 (5.1)	23.1 (8.5)	23.8 (6)	24.5 (4.9)	18.1 (3.2)	18.7 (4.2)	19.5 (5.6)	20.3 (3.9)	17.9 (5.9)	21.3 (5.5)
12 months	25 (10.7)	24.2 (4.7)	26.1 (6.4)	20.2 (6)	22.1 (4)	22.8 (3.8)	17.2 (2.9)	18.3 (3.8)	18.7 (5.4)	20.6 (4.6)	20.6 (6.2)	20.4 (3.5)
**Total fiber, g/1000 kcal**												
Baseline	11.8 (4)	9.2 (3.3)	9 (2.7)	10 (2.9)	11.6 (2.9)	11.1 (2.8)	12.5 (6.2)	9.3 (2.7)	10.1 (3.1)	14 (4.3)	11.7 (2.7)	10.3 (4)
3 months	9.6 (3.8)	8.7 (2.9)	8.8 (3)	9.7 (3.6)	24.1 (45.9)	11.9 (5.1)	20 (5.9)	17.2 (5.1)	25.6 (10)	13.8 (2.4)	16.6 (4.7)	17 (4.4)
6 months	9.7 (4.8)	8.3 (3.2)	9.6 (4.9)	11.5 (5.3)	10.8 (2.7)	13.3 (3.3)	15.5 (2.4)	15.3 (4.8)	18.1 (9.7)	15.6 (4.5)	15.3 (6)	15.3 (5.6)
12 months	13.5 (4.1)	10.8 (2.5)	10.6 (4.5)	10.8 (4.2)	12.5 (5.3)	11 (5.6)	14.3 (2.9)	13.2 (5.5)	17.5 (8.3)	13.9 (3.1)	12.1 (5.3)	13.6 (4.2)
**Soluble fiber, g/1000 kcal**												
Baseline	3 (0.7)	2.5 (0.7)	2.9 (0.9)	2.9 (0.9)	3.5 (1)	3.7 (1)	4 (2.2)	3.2 (1.1)	2.9 (0.9)	4.1 (1.5)	3.5 (0.7)	3.2 (1.3)
3 months	2.1 (1.2)	2.5 (1)	2.6 (1.7)	3.3 (1.3)	2.8 (1)	3.3 (2.3)	5.3 (2)	4.8 (1.3)	6 (2.3)	4 (0.7)	4.8 (2)	5.3 (1.6)
6 months	2.1 (1.1)	2.8 (1.3)	2.5 (1.6)	4.1 (2.6)	3.1 (1)	3.9 (1.6)	5 (1.7)	4.8 (1.7)	4.4 (2.6)	4.4 (1.4)	3.9 (1.4)	4.5 (1.7)
12 months	3.2 (0.9)	2.9 (0.8)	2.7 (1.4)	3.4 (1.4)	3.5 (1.2)	3.3 (2.5)	4.5 (1.2)	4 (1.4)	4.5 (2.1)	4.3 (1.2)	3.5 (0.9)	3.8 (0.9)
**Insoluble fiber, g/1000 kcal**												
Baseline	8.8 (3.7)	6.7 (3)	6 (2.2)	7 (2.5)	8 (2.4)	7.4 (1.9)	8.4 (4.3)	6 (1.7)	7.1 (2.4)	9.4 (2.9)	8.2 (2.3)	7.1 (2.8)
3 months	7.5 (2.8)	6.1 (2.4)	6.2 (2.3)	6.4 (2.5)	8.4 (3.5)	8.7 (3.8)	14.6 (4.3)	12.4 (4.3)	19.4 (8.2)	9.9 (2.3)	11.7 (2.9)	11.6 (3.9)
6 months	7.4 (4.1)	5.4 (2.4)	6.8 (3.6)	7.2 (2.9)	7.7 (2)	9.4 (3.1)	11.1 (2.1)	10.5 (3.7)	13.7 (7.5)	11.2 (3.7)	11.3 (4.9)	10.8 (4.2)
12 months	10.2 (3.7)	7.9 (2.2)	7.8 (3.8)	7.4 (3.2)	8.9 (4.8)	7.6 (3.3)	9.7 (2.2)	9.2 (4.4)	13 (6.3)	9.6 (2.5)	8.6 (4.4)	9.7 (3.5)
**Dietary adherence[1], % kcals (SD)**	70.8 (10.8)	78.3 (9.8)	81.2 (8.6)	64.1 (8.7)	68.4 (6.3)	78.6 (6.6)	73.6 (5.5)	74.3 (5)	76.8 (7.2)	68.4 (5.1)	71.6 (8)	77.7 (6.6)
**Dietary change[2], % kcals (SD)**	17.7 (9.6)	18 (11.4)	27.8 (7.8)	10.5 (12)	15.6 (8.1)	21.1 (6.8)	8 (7)	11.3 (6.9)	16.4 (6.1)	5.7 (4.7)	9.2 (7.6)	10.9 (5.6)

[1] Portion of diet from non-restricted foods, averaged over 3-, 6-, and 12-month dietary recalls. Carbohydrate restriction for subjects on low-carb diet; fat restriction for subjects on low-fat diet.

[2] Decrease in restricted foods from baseline, using average of 3-, 6-, and 12-month dietary recalls.

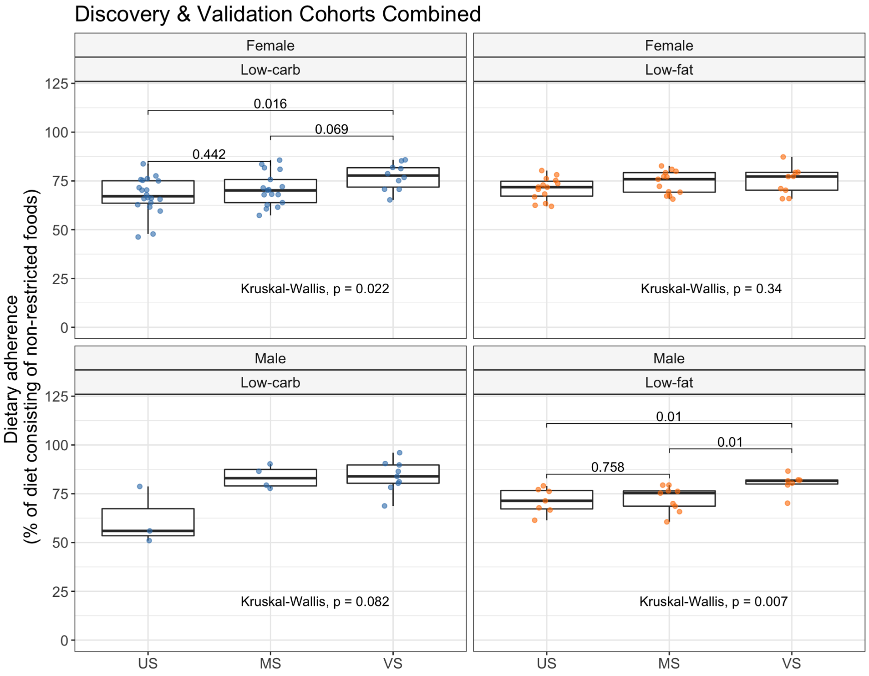

(a) Dietary adherence

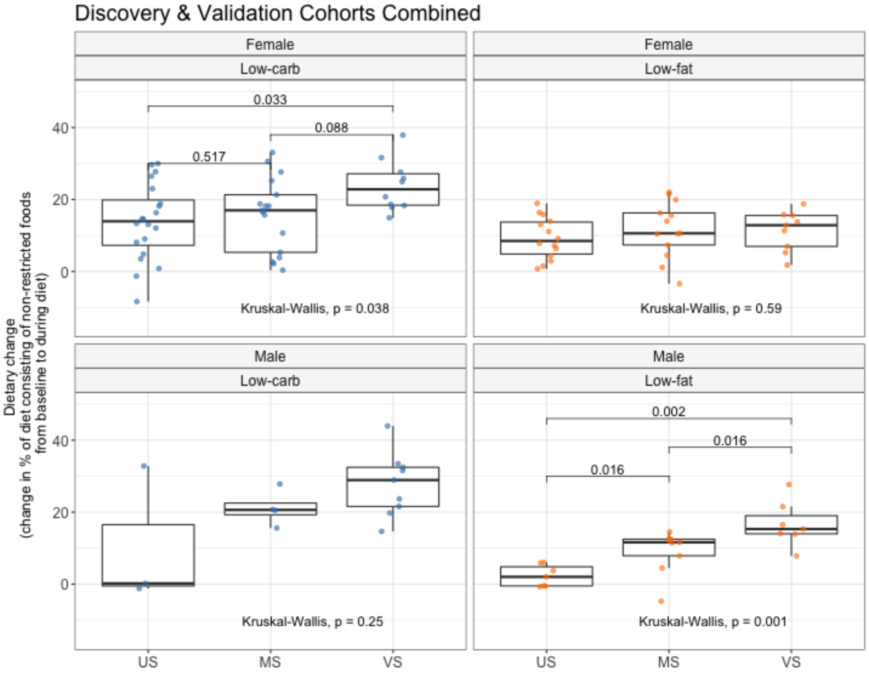

(b) Dietary change

Supplementary Figure S1: **Dietary adherence and dietary change** between 12-month weight loss success groups, evaluated by sex and diet. US – Unsuccessful, $< 3\%$ weight loss; MS – Moderately successful, $3 - 10\%$ weight loss; VS – Very successful, $> 10\%$ weight loss. A Kruskal-Wallis test was used to determine if there was a difference between groups (P-values shown in bottom right of each plot) and where significant differences between groups were found, P-values are shown for pairwise Wilcoxon rank sum test between groups after adjustment for multiple corrections with Benjamimi Hochberg procedure. The analysis was done for dietary adherence, i.e. the percentage of total kcals coming from non-restricted foods (a), and also dietary change, i.e. decrease in percentage of total daily kcals consumed from restricted foods (fats/carbs) from baseline to while on the diet (b). See Methods for more details on dietary adherence and dietary change definitions.

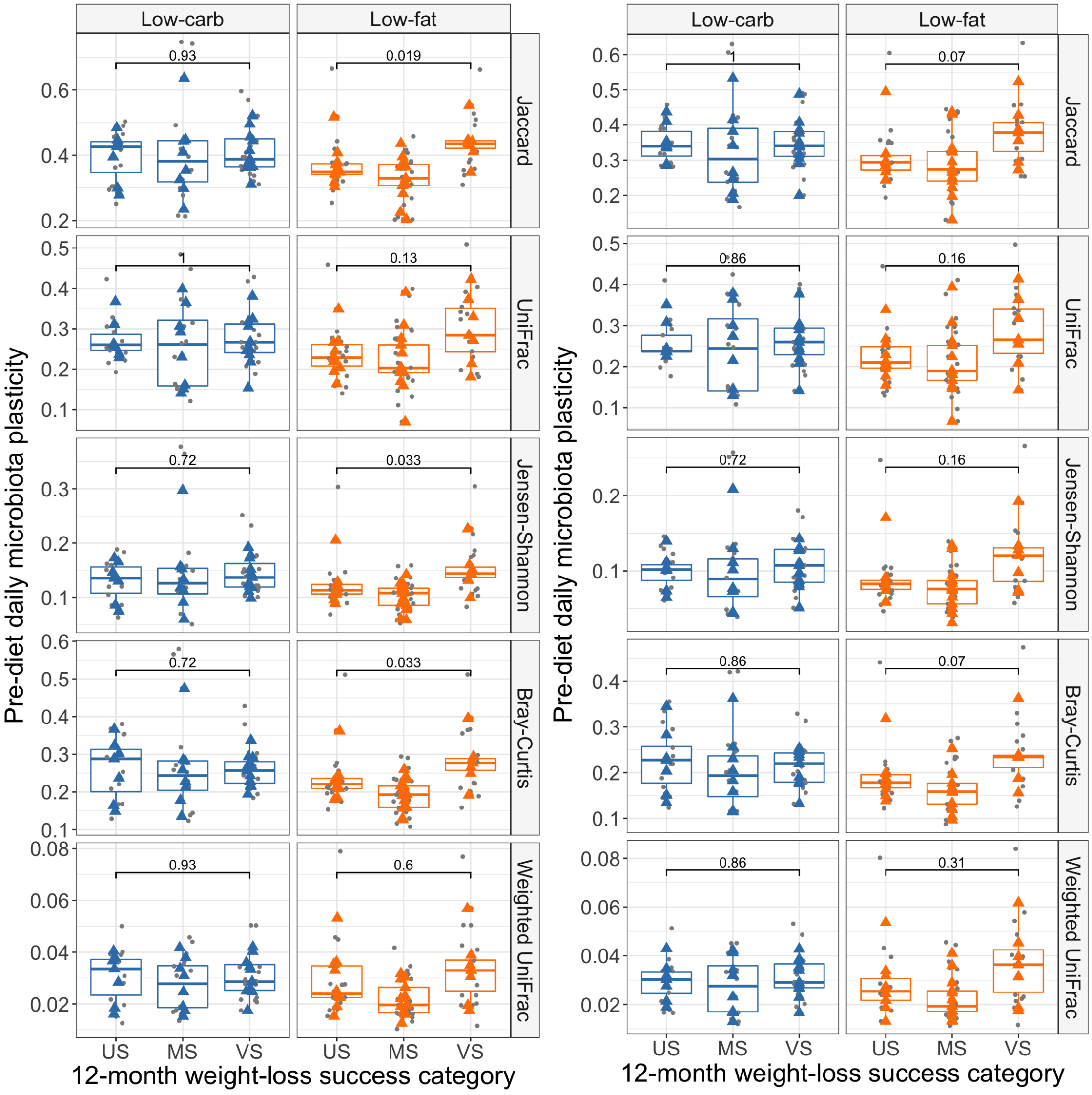

(a) ASV analysis

(b) ASV-cluster analysis

Supplementary Figure S2: **Day-to-day plasticity (β-diversity) between pre-diet fecal samples** from subjects in the discovery cohort, grouped by 12-month weight loss success. Jaccard, unweighted UniFrac, Jensen-Shannon-Divergence, Bray-Curtis, and weighted UniFrac distances are shown for low-carb diet (left panels, in blue) and low-fat diet (right panels, in orange). Grey points indicate computed pairwise dissimilarities between samples; colored (low-carb and low-fat) points indicate the average sample-to-sample dissimilarity for each subject.US – Unsuccessful, $<3\%$ weight loss; MS – Moderately successful, $3-10\%$ weight loss; VS – Very successful, $>10\%$ weight loss. P-values shown for Wilcoxon rank-sum test comparing US and VS groups. The analysis was done at the level of individual ASVs (a) and also ASV-clusters (b).

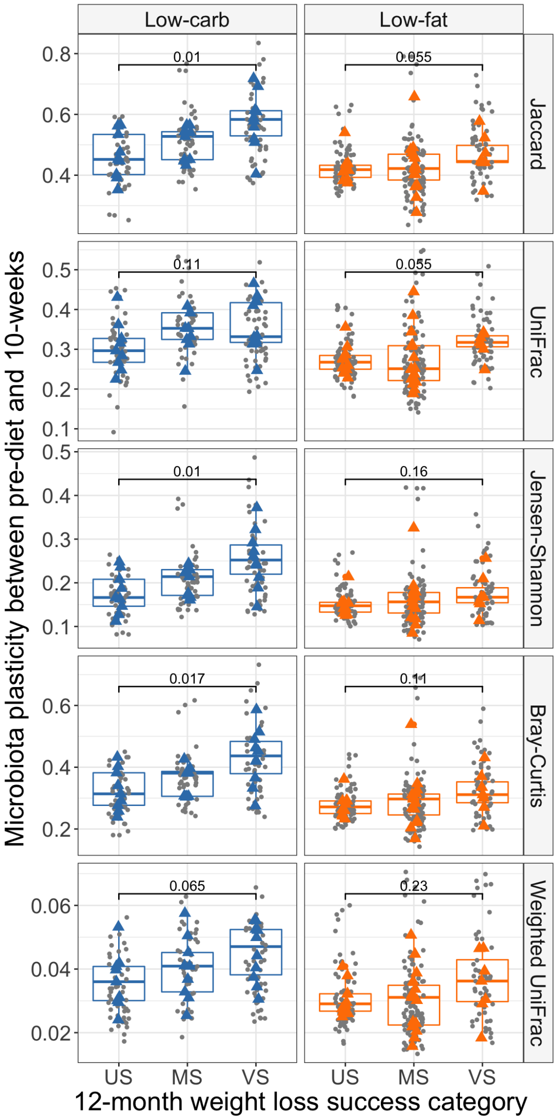

(a) ASV analysis

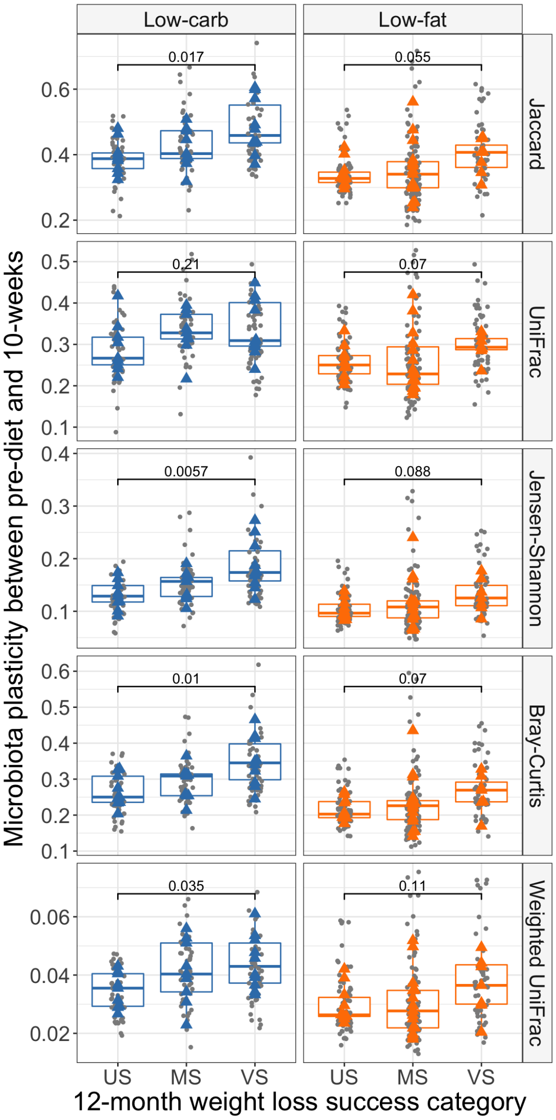

(b) ASV-cluster analysis

Supplementary Figure S3: **Microbial community composition shift over ten weeks, and in response to dietary intervention**. β-diversity between pre-diet (baseline) and post-diet samples (taken at 10 weeks after initiation of the dietary intervention) from each subject was computed using Jaccard, unweighted UniFrac, Jensen-Shannon-Divergence, Bray-Curtis, and weighted UniFrac distances. Results shown for discovery cohort subjects on a low-carb (left panels, in blue) or low-fat (right panels, in orange) diet. Grey points indicate each computed pairwise dissimilarity between samples; colored points correspond to the average baseline-to-10-week plasticity for each subject. US – Unsuccessful, $< 3\%$ weight loss; MS – Moderately successful, 3-10% weight loss; VS – Very successful, $\geq 10\%$ weight loss. P-values shown for Wilcoxon rank-sum test comparing US and VS groups. The analysis was done at the level of individual ASVs (a) and also ASV-clusters (b).

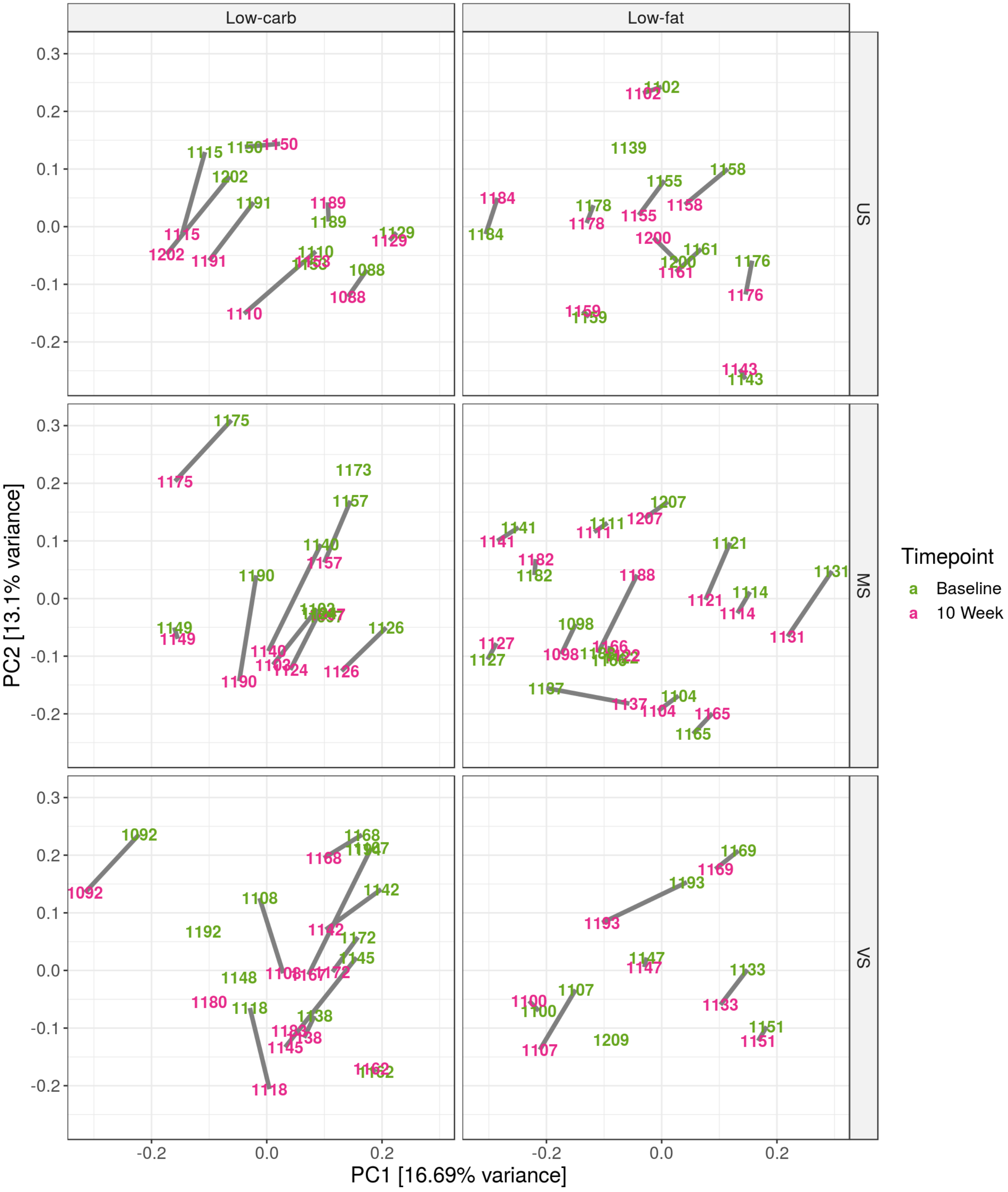

Supplementary Figure S4: **Microbiome shift in response to dietary intervention.** Principal coordinates analysis shows subject centroids (coordinates averaged over sample replicates) before (green) and 10 weeks after the start of the intervention (purple) for low-carb (*left*) and low-fat (*right*) diet. Data points are labeled with corresponding unique subject ID. Vertical panels correspond to weight loss success categories – very successful (VS), moderately successful (MS) and unsuccessful (US).

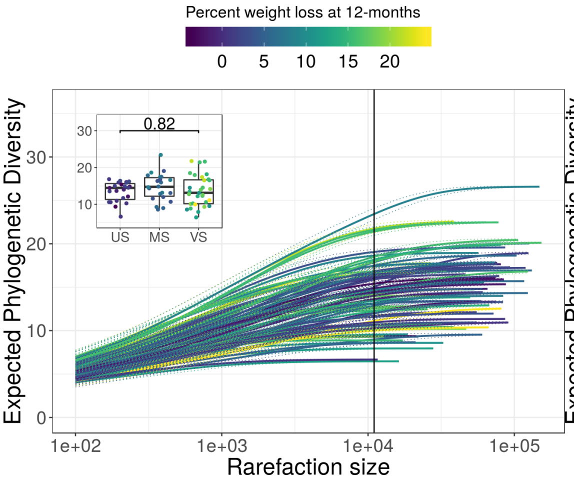

(a) Discovery Cohort: Low-carb

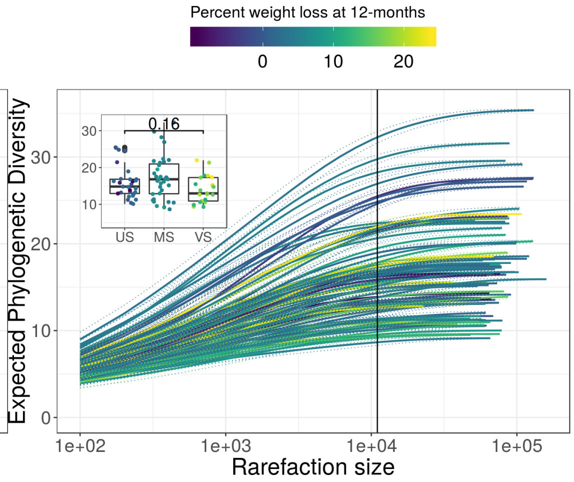

(b) Discovery Cohort: Low-fat

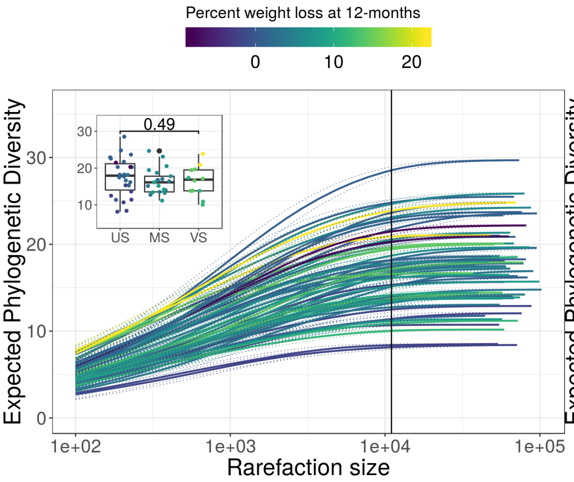

(c) Validation Cohort: Low-carb

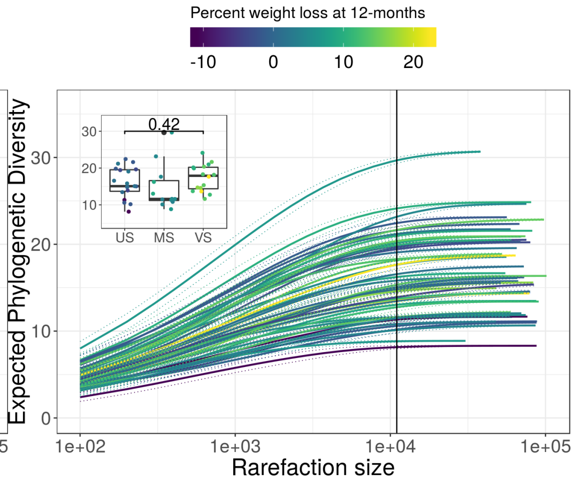

(d) Validation Cohort: Low-fat

Supplementary Figure S5: **Phylogenetic α-diversity in pre-diet samples.** Rarefaction curves for low-carb (*left*) and low-fat (*right*) diet, separated by discovery (*top*) and validation (*bottom*) cohorts. Curves were computed using the methods described in [1]. Inset boxplots report non-significant differences in α-diversity (at rarefaction level = 11,000 reads) between subjects from the VS and US 12-month weight loss success categories.

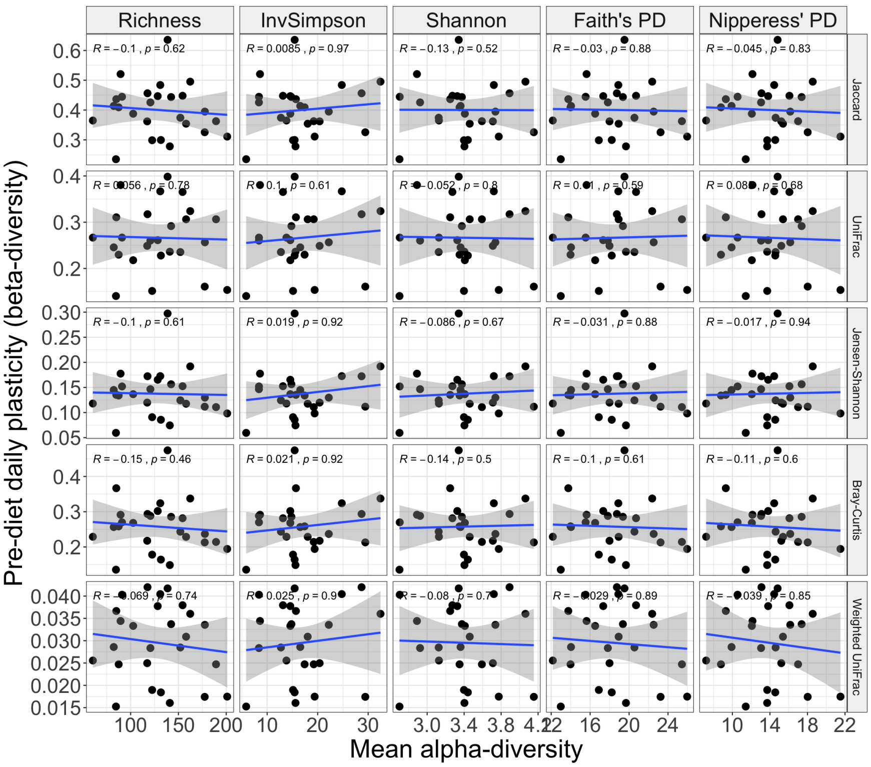

(a) Low-carb

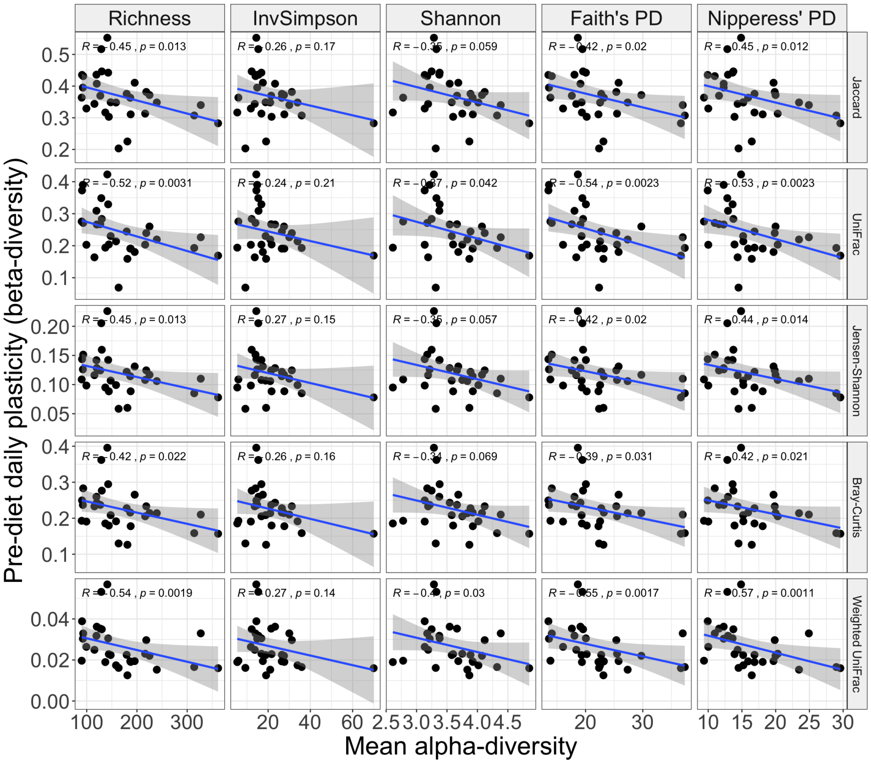

(b) Low-fat

Supplementary Figure S6: **Pre-diet plasticity (β-diversity) negatively correlated with α-diversity** for subjects on the low-fat diet. Points represent individual subjects. Mean pre-diet bacterial community α-diversity was calculated using up to three samples for each subject and plasticity measures included the average of consecutive daily samples. Spearman's rank correlation coefficient (R) and corresponding p-value are shown. Note that both the α- and β-diversity measures were computed for samples obtaine before any dietary intervention.

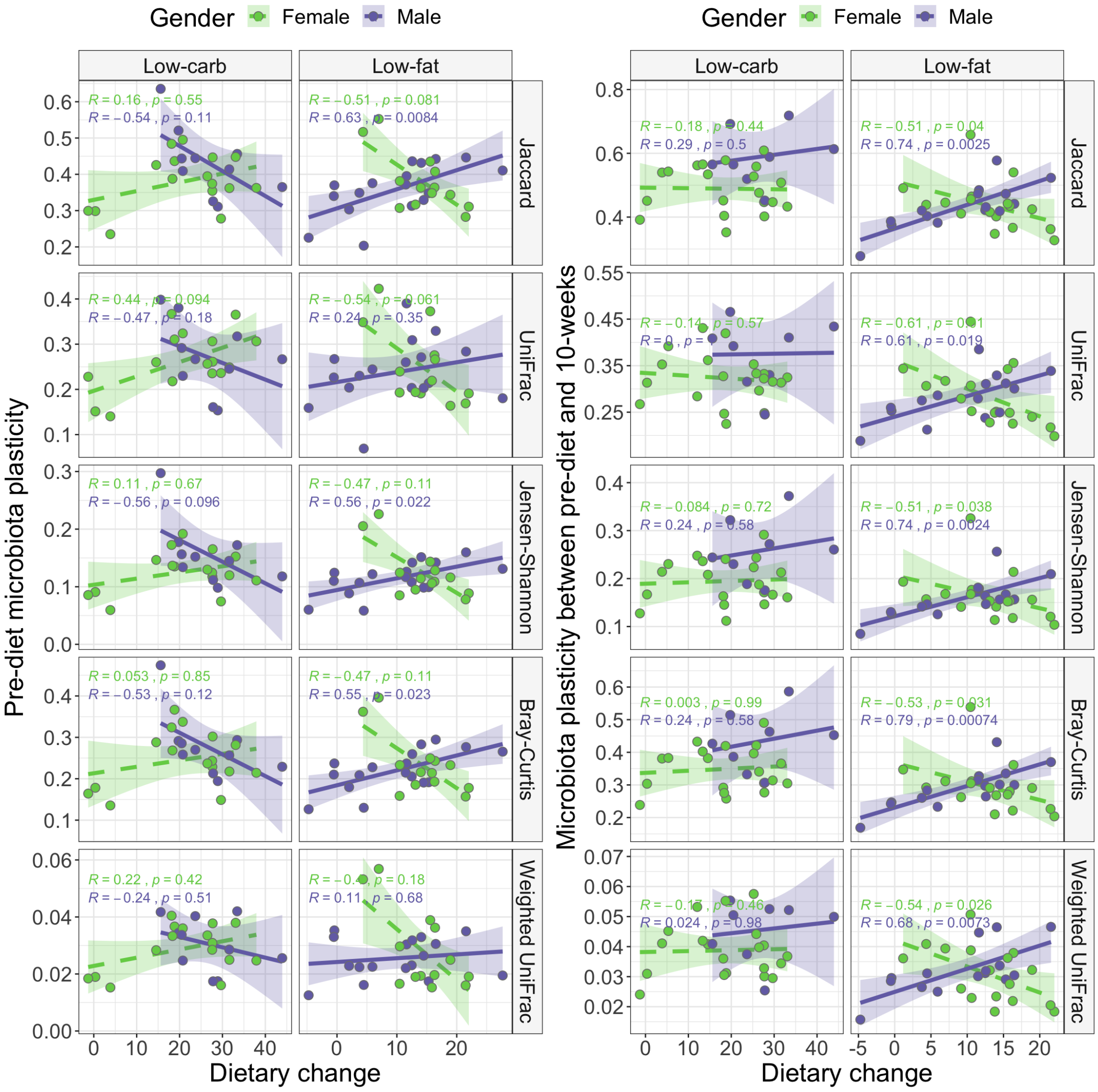

(a) (b)

Supplementary Figure S7: **Correlations between dietary change and microbial community pre-diet baseline plasticity (a) and plasticity in response to 10 weeks of dietary intervention (b)**. Spearman's rank correlations between dietary change and plasticity (measured via various distance metrics) between daily pre-diet samples (a) and between pre-diet and 10-week samples (b) are shown for low-carb (left panels) and low-fat (right panels) diets. Male (purple) and female (green) subjects show opposite correlations in many cases.

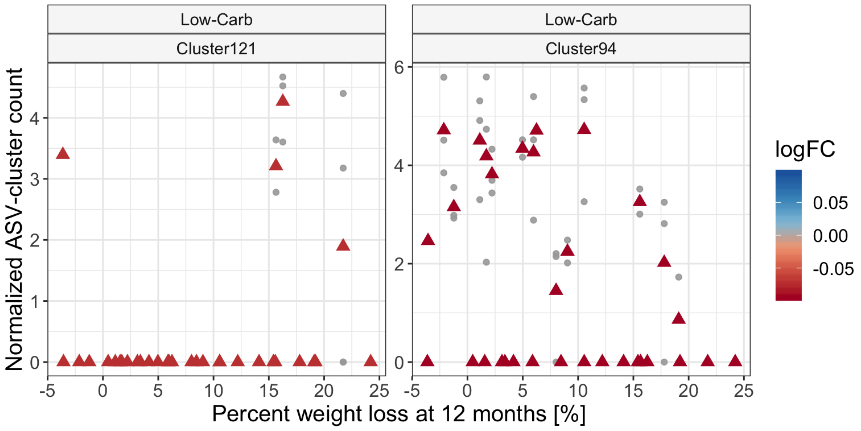

(a)

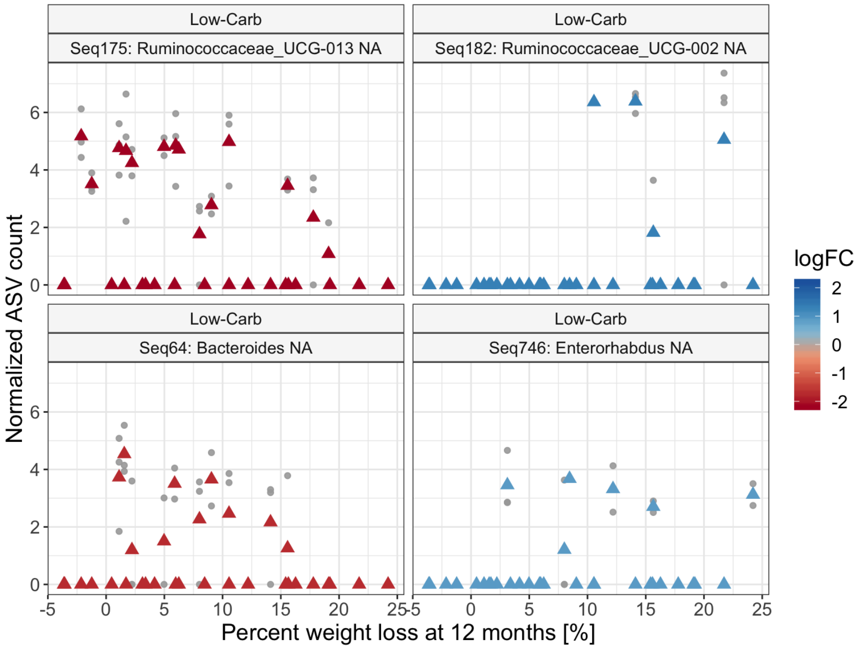

(b)

Supplementary Figure S8: **Taxa identified differentially abundant for subjects on low-carb diet.** Analyses were conducted with ASV-clusters testing for difference in abundance across a continuous response variable – percent weight loss (a) and also with individual ASVs and tested for contrast between categorical weight loss groups: VS compared to US subjects. Note: Cluster94 contains Seq175, as can be seen in the similarity of plots. Individual ASVs and ASV-clusters were normalized and asinh-transformed for variance stabilization prior to analysis; the normalized, transformed values are shown on the y-axis. Grey points represent individual samples and triangles represent the mean value for each subject. No taxa were found differentially abundant on the low-fat diet for either analysis.

References

[1] David A Nipperess and Frederick A Matsen IV. The mean and variance of phylogenetic diversity under rarefaction. Methods Ecol Evol, 4(6):566–572, 2013.
